# Synthesis and biological activity of methylated derivatives of the *Pseudomonas* metabolites HHQ, HQNO and PQS

**DOI:** 10.3762/bjoc.15.18

**Published:** 2019-01-21

**Authors:** Sven Thierbach, Max Wienhold, Susanne Fetzner, Ulrich Hennecke

**Affiliations:** 1 Institute for Molecular Microbiology and Biotechnology, University of Münster, Corrensstr. 3, 48149 Münster, Germany; 2 Organic Chemistry Institute, University of Münster, Corrensstr. 40, 48149 Münster, Germany; 3 Organic Chemistry Research Group, Departments of Chemistry and Bioengineering Sciences, Vrije Universiteit Brussel, Pleinlaan 2, 1050 Brussel, Belgium

**Keywords:** antibiotic acitivity, methylation, *Pseudomonas aeruginosa*, quinolones, quorum sensing

## Abstract

Selectively methylated analogues of naturally occurring 2-heptyl-4(1*H*)-quinolones, which are alkaloids common within the Rutaceae family and moreover are associated with quorum sensing and virulence of the human pathogen *Pseudomonas aeruginosa*, have been prepared. While the synthesis by direct methylation was successful for 3-unsubstituted 2-heptyl-4(1*H*)-quinolones, methylated derivatives of the *Pseudomonas* quinolone signal (PQS) were synthesized from 3-iodinated quinolones by methylation and iodine–metal exchange/oxidation. The two *N*- and *O*-methylated derivatives of the PQS showed strong quorum sensing activity comparable to that of PQS itself. *Staphylococcus aureus*, another pathogenic bacterium often co-occurring with *P. aeruginosa* especially in the lung of cystic fibrosis patients, was inhibited in planktonic growth and cellular respiration by the 4-*O*-methylated derivatives of HQNO and HHQ, respectively.

## Introduction

2-Alkyl-4(1*H*)-quinolones (AQs) have been identified as natural products produced by higher plants of the Rutaceae family as well as by some microorganisms including *Alteromonas*, *Burkholderia* and *Pseudomonas* species [1–9]. Plant-derived AQs occur with alkyl chains of different lengths, branches and unsaturation and can be *O*- or *N*-methylated [1–3]. In the opportunistic pathogen *Pseudomonas aeruginosa*, AQ derivatives with heptyl or nonyl side chains are prevalent [3,7–9]. 2-Heptyl-3-hydroxy-4(1*H*)-quinolone (*Pseudomonas* quinolone signal, PQS) and its biosynthetic precursor 2-heptyl-4(1*H*)-quinolone (HHQ, **1**) are important signaling molecules involved in quorum sensing and as such play an important role in virulence regulation [3,10–12]. Another metabolite from the AQ biosynthesis pathway of *P. aeruginosa* is 2-heptyl-1-hydroxy-4(1*H*)-quinolone (generally referred to as 2-heptyl-4-hydroxyquinoline *N*-oxide, HQNO), which is not active in quorum sensing, but interferes with the respiratory electron transport via inhibition of the cytochrome *bc*_1_ complex and moreover inhibits pyrimidine biosynthesis [9,12–14]. Therefore, HQNO is toxic to many organisms. In dual-species co-cultures of *P. aeruginosa* and *Staphylococcus aureus*, which model interactions relevant to patients with cystic fibrosis, HQNO is a major factor for killing of *S. aureus* [15]. While HQNO seems to be generally toxic to Gram-positive bacteria, we could show that some bacteria are able to transform HQNO into less toxic metabolites [16]. For example, *Mycobacterium abscessus*, which like *P. aeruginosa* and *S. aureus* can occur in the lung of cystic fibrosis patients [17–19], is able to methylate HQNO to give 2-heptyl-1-methoxy-4(1*H*)-quinolone (HMOQ). HMOQ is a significantly less efficient inhibitor of the respiratory chain cytochromes in *Mycobacteria* than HQNO and therefore the methylation of HQNO can be seen as detoxification strategy [16].

Considering the natural occurrence of *N*- as well as *O*-methylated AQs in plants and the use of methylation of HQNO in *Mycobacteria* as detoxification reaction, we decided to investigate the effect of methylation of AQs on their biological properties more systematically. To this end, we prepared selectively methylated derivatives of the most important heptyl-AQs including HHQ, HQNO and PQS derivatives. These synthetic compounds were tested for their antimicrobial properties against *S. aureus* and for their quorum sensing activity.

## Results and Discussion

### Synthesis of methylated heptyl-AQs

**Scheme 1:**
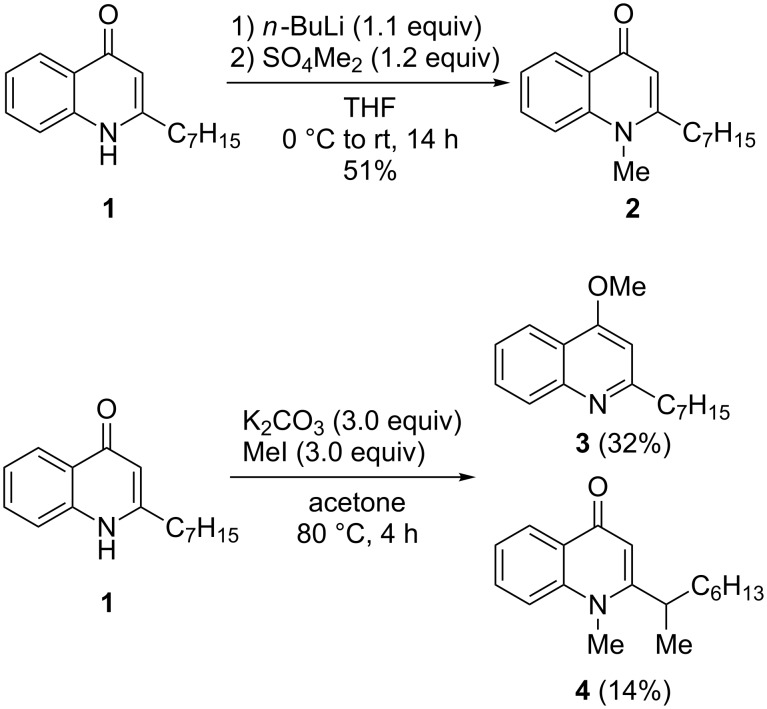
Methylation of HHQ (**1**).

The synthesis of methylated AQ derivatives started from HHQ (**1**), which is conveniently available via Conrad–Limpach reaction in large quantities [20–21]. Upon treatment of HHQ (**1**) with *n*-butyllithium and dimethyl sulfate mainly *N*-methylation was observed accompanied by a small amount of the *N*,*O*-dimethylquinolonium ion. Pure NMe-HHQ (**2**) was obtained in moderate yield of 51% by column chromatography (Scheme 1).

Selective *O*-methylation of an AQ has been reported using diazomethane [1]. To avoid explosive diazomethane, methylation of HHQ with trimethylsilyldiazomethane (TMS-DA) in methanol was carried out, which indeed provided selectively OMe-HHQ (**3**). However, even using a large excess of TMS-DA low conversion and yields of less than 20% OMe-HHQ (**3**) were observed. Addition of HBF_4_ did improve the reactivity [22], but instead of the desired **3** dimethylation to give the *N*,*O*-dimethylquinolonium ion was observed. Alternatively, HHQ was methylated using MeI and K_2_CO_3_ [23]. This procedure provided a mixture of *O*- and *N*-methylated products with a slight preference for OMe-HHQ (**3**). OMe-HHQ (**3**) could be easily separated from the *N*-methylation product by column chromatography and could be isolated in 32% yield. Surprisingly, the *N*-methylated product under these conditions was not NMe-HHQ (**2**), but instead a second methylation in the benzylic position had occurred to give *N*-methyl-2-(1-methylheptyl)-4(1*H*)-quinolone (**4**), which could be isolated in 14% yield.

**Scheme 2:**
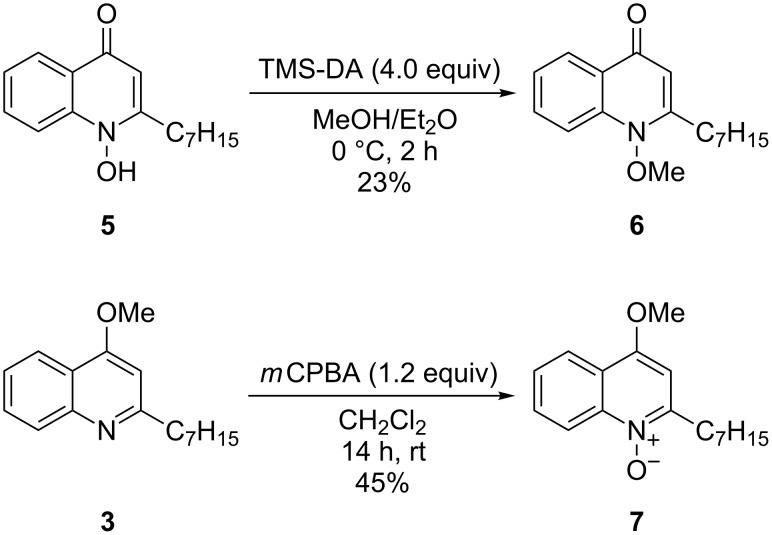
Synthesis of methylated HQNO derivatives.

For the synthesis of methylated HQNO derivatives, HHQ (**1**) was converted to HQNO (**5**) using a literature procedure [24], which was slightly modified to provide HQNO in 69% yield over three steps without purification by column chromatography (see Supporting Information File 1 for details). Selective methylation of one of the *O*-atoms of HQNO was attempted using various methods. Treatment of HQNO (**5**) in MeOH/Et_2_O with TMS-DA resulted in selective methylation of the N–O oxygen atom to provide 1-methoxy-2-heptyl-4(1*H*)-quinolone (HMOQ (**6**), Scheme 2) [16]. The methylation was highly selective and no methylation at the 4-position oxygen atom was observed, however, conversion and, therefore, yields remained low even with a large excess of reagent. Addition of HBF_4_ did not improve the yield, but led to side products, which proved to be difficult to remove.

Several attempts were made to obtain the 4-*O*-methylated HQNO by direct methylation of HQNO (**5**), however, treatment of HQNO with strong bases such as NaH followed by a methylating agent (MeI) did not provide methylated HQNO derivatives, but complex mixtures containing decomposition products including HHQ (**1**) and methylated HHQ derivatives. The N–O bond appeared to be unstable under these alkylation conditions. Therefore, the synthesis of the 4-methoxy derivative was carried out starting from OMe-HHQ (**3**). Upon treatment of **3** with *m*CPBA the compound was cleanly oxidized to 4OMe-HQNO (**7**, Scheme 2). Purification by column chromatography provided the highly polar compound **7** in acceptable yield (45%).

**Scheme 3:**
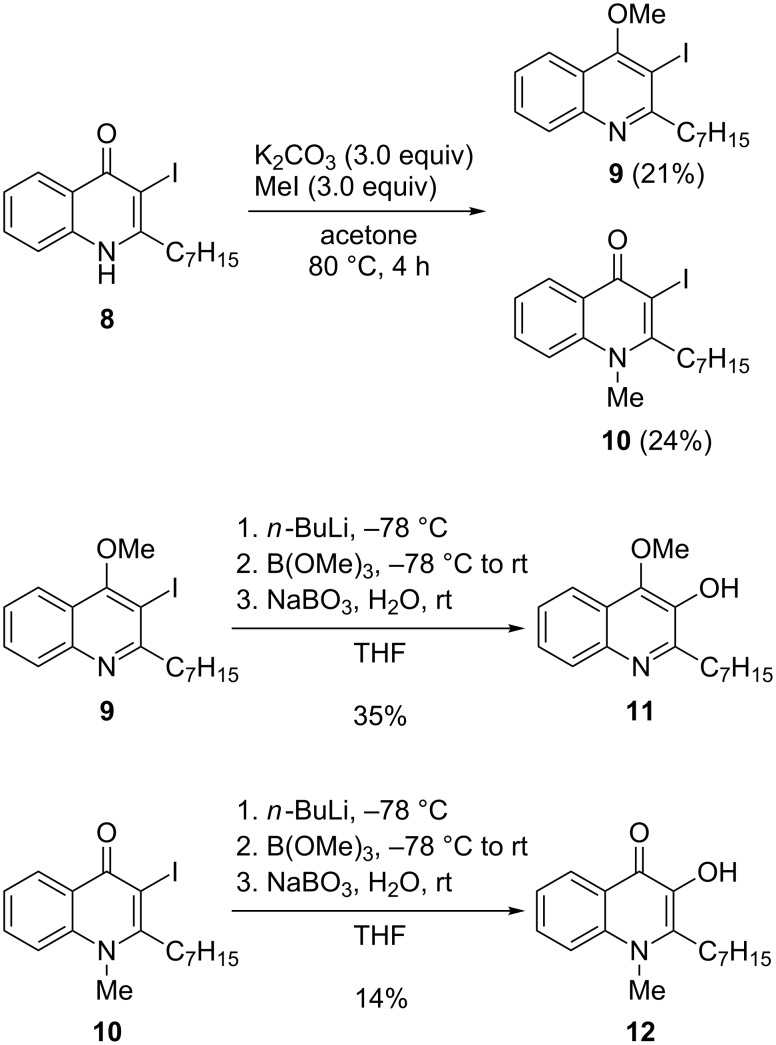
Synthesis of methylated PQS derivatives.

Finally, the synthesis of methylated PQS derivatives was carried out. Initially, the direct conversion of OMe-HHQ (**3**) into 4OMe-PQS (**11**) was attempted. However, the standard methodology for the conversion of HHQ into PQS such as formylation followed by oxidation failed when applied to OMe-HHQ (**3**). Alternatively, *ortho*-metalation next to the methoxy group of OMe-HHQ (**3**) followed by borylation/oxidation was investigated. Several trials using different lithium bases failed and only small amounts of oxidation products in the benzylic position could be observed. Instead of direct *ortho*-metalation, a strategy based on halogen–lithium exchange proved to be more suitable. To this end, HHQ (**1**) was converted into 3I-HHQ (**8**) following a literature procedure [21]. 3I-HHQ (**8**) was then methylated using the MeI/K_2_CO_3_ conditions to give a mixture of OMe-3I-HHQ (**9**, 21%) and NMe-3I-HHQ (**10**, 24%, Scheme 3). Interestingly, in the case of 3I-HHQ (**8**) the ratio of *N*-methylation versus *O*-methylation was almost 1:1 and no further methylation of **10** in the benzylic position was observed. With the methylated compounds **9** and **10** in hand, the halogen-lithium exchange was attempted. Treatment of **9** with *n*-butyllithium at −78 °C followed by the addition of B(OMe)_3_ and finally in situ oxidation using sodium perborate provided the desired 4OMe-PQS derivative **11** in moderate yield (35%, Scheme 3). The same procedure could be applied to NMe-3I-HHQ (**10**), however, the resulting NMe-PQS derivative **12** was only isolated in low yield (14%). Presumably, this compound is not too stable during the final oxidation step as significant amounts of oxidative side products including *N*-methylisatin were obtained, which also impeded the isolation of the desired product **12**. Nevertheless, enough material could be obtained to enable biological investigations on this compound.

### Growth inhibition of *S. aureus*

**Figure 1:**
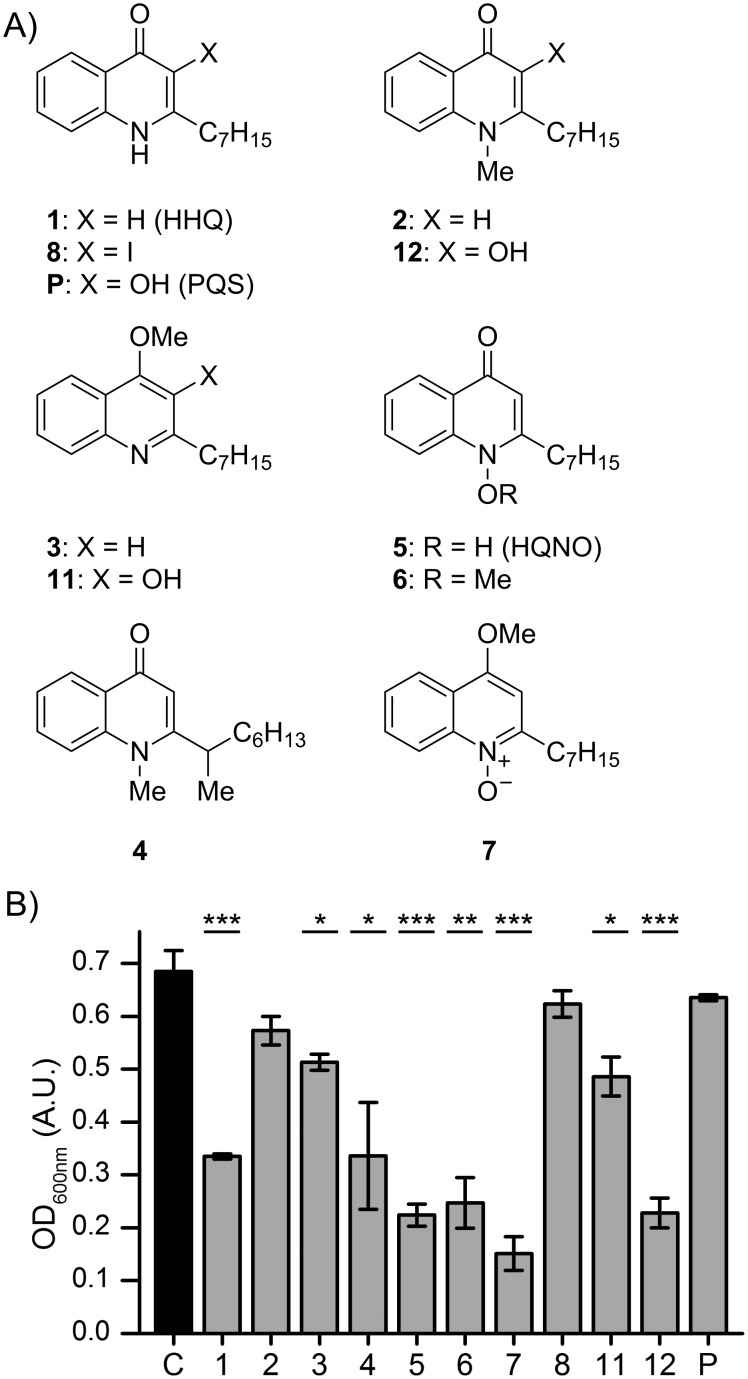
Overview of AQ compounds (A), and effect of AQs on the growth of *S. aureus* Newman (B). 24-Well plates were inoculated at an optical density (OD_600 nm_) of 0.1 and supplemented with 100 µM of the indicated AQs (**1**–**12**), PQS (P) or an equal amount of DMSO as the control (C). Black and grey bars show the mean optical density after 4 hours of cultivation at 37 °C of 3 biological replicates. Error bars represent the standard error. One-way ANOVA was used for statistical analysis, and significant differences compared to the control are indicated (* *P* < 0.05, ** *P* < 0.01, *** *P* < 0.001).

To get a better understanding of the influence of methylation on the antibiotic properties of AQ compounds, their effect on the growth of *S. aureus* Newman was investigated (Figure 1). To be able to compare the growth in the presence of the only weakly antibiotic HHQ derivatives, growth experiments were conducted at a concentration of 100 µM. Planktonic cultures of *P. aeruginosa* in LB medium were reported to contain up to 50 µM PQS and up to 70 µM HQNO [25]. As observed previously, HQNO (**5**) inhibited the growth of *S. aureus* significantly, while HHQ (**1**) had only a moderate effect. Interestingly, the methylated HQNO derivatives **6** and **7** were either equally potent as HQNO or, in the case of **7**, even led to a stronger growth reduction. While most of HHQ and PQS derivatives showed no or only very weak effects on the growth of *S. aureus*, NMe-PQS (**12**) caused a similar growth inhibition as HQNO. However, the AQs did not significantly affect the formation of static biofilms of *S. aureus* under the conditions tested (see Figure S1, Supporting Information File 1).

**Figure 2:**
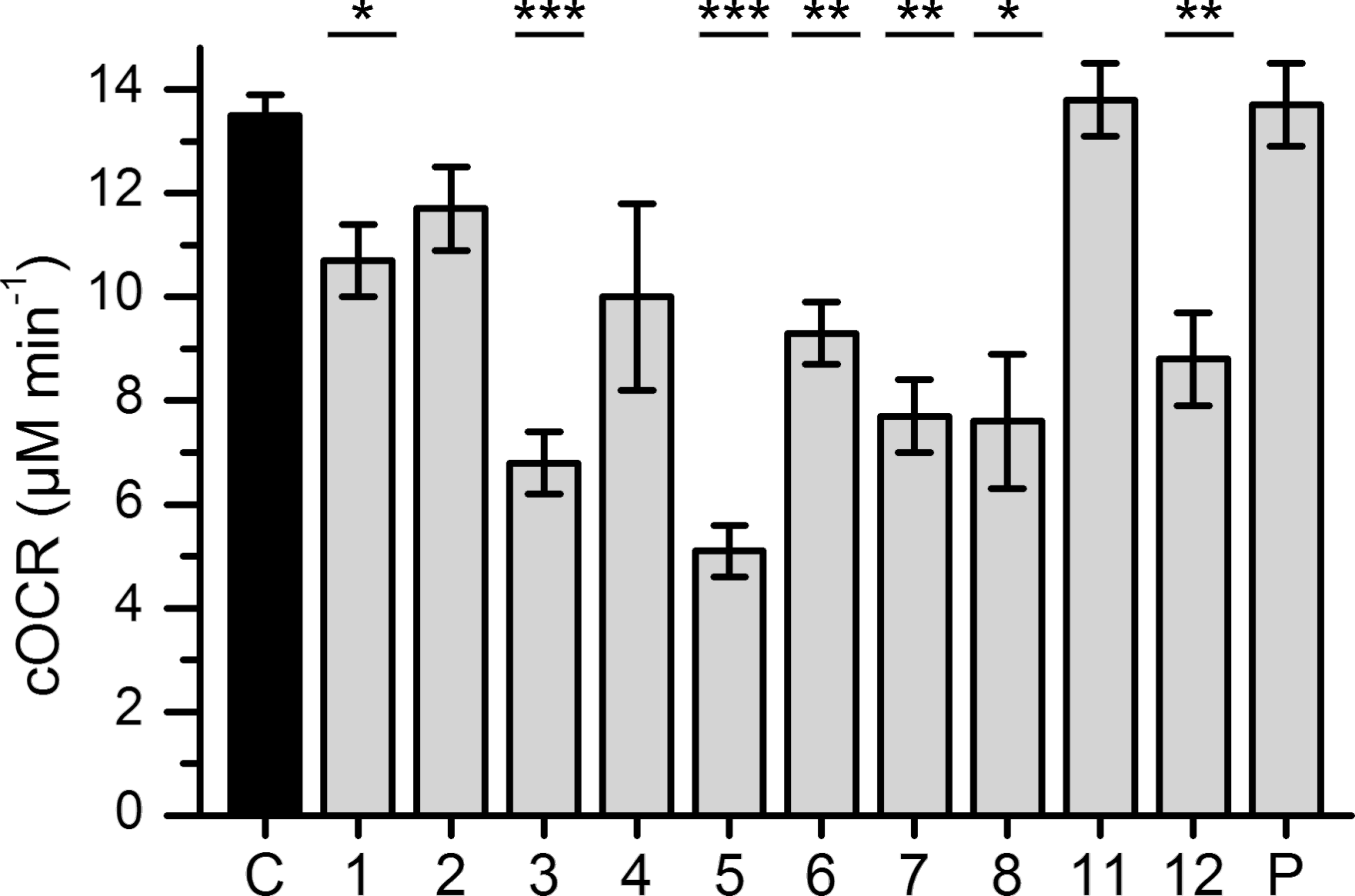
Inhibition of cellular O_2_ consumption rate (cOCR) of *S. aureus* Newman. Cell suspensions (OD_600 nm_ of 0.5) were incubated with 100 µM of the indicated AQs (**1**–**12**), PQS (P) or an equal amount of DMSO as the control (C). Black and grey bars show the mean cOCR of three biological replicates. Error bars represent the standard error. One-way ANOVA was used for statistical analysis, and significant differences compared to the control are indicated (* *P* < 0.05, ** *P* < 0.01, *** *P* < 0.001).

HQNO is known to inhibit complex III of the respiratory chain and therefore leads to a measurable reduction in O_2_ consumption by cells of *S. aureus* [16]. The investigation of the O_2_-consumption rates of *S. aureus* in response to AQs revealed that among the compounds tested, HQNO (**5**) had the strongest effect (Figure 2). The 4-*O*-methylated HHQ and HQNO derivatives **3** and **7**, but not the respective PQS derivative **11**, also inhibited O_2_ consumption. In contrast, only weak to moderate inhibition was observed with derivatives methylated either at the nitrogen atom (**2**, **4** and **12**) or at the N–O oxygen atom (**6**).

### Quorum sensing activity

**Figure 3:**
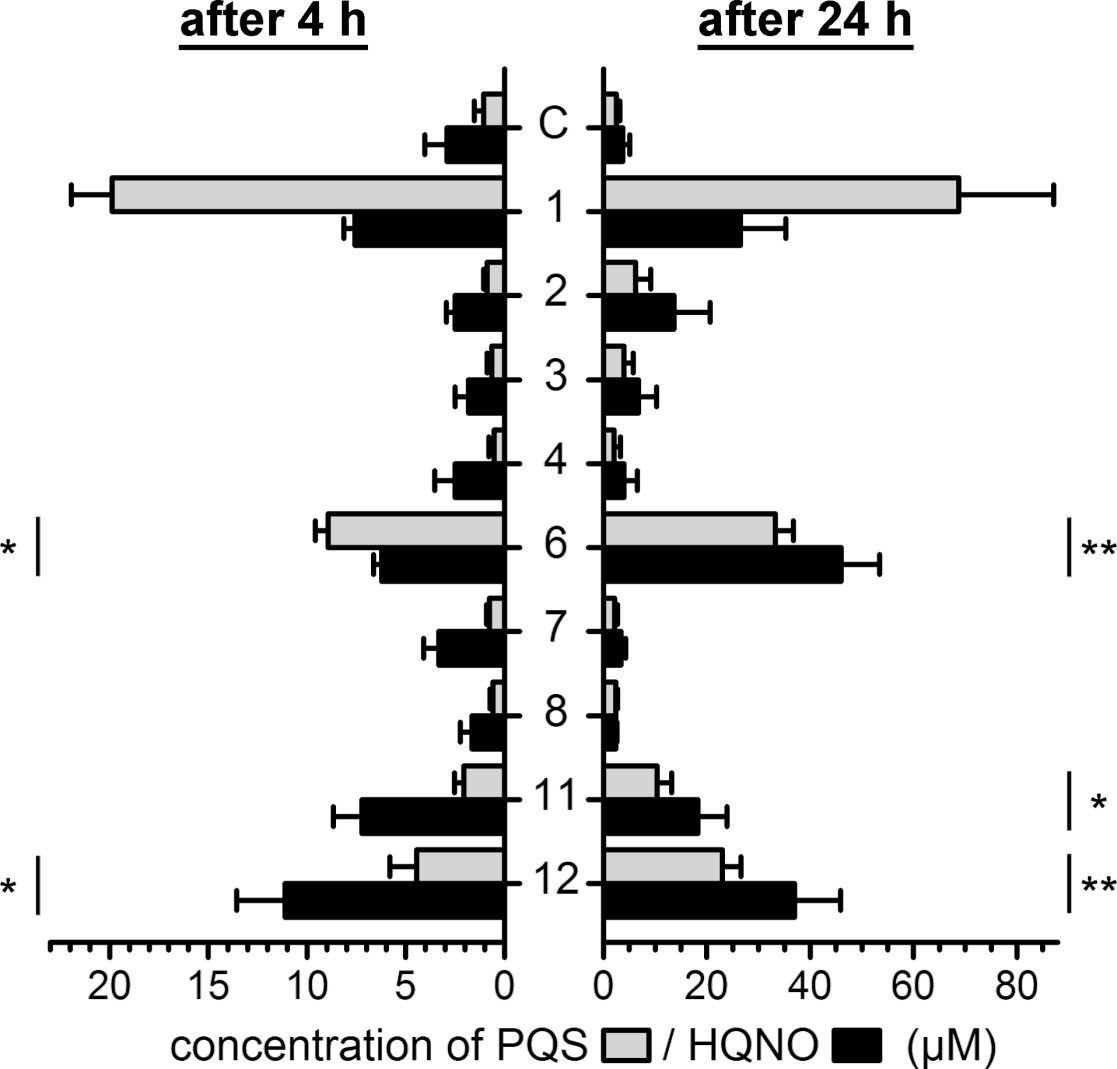
Impact on AQ quorum sensing by the newly synthesized AQ derivatives. Cultures of *P. aeruginosa* PAO1 were supplemented with 100 µM of AQs (**1**–**12**) or an equal amount of DMSO as the control (C), and the concentrations of PQS (grey bars) and HQNO (black bars), as readout of the signal-induced upregulation of AQ biosynthesis, were measured at an early (after 4 hours; left) and a late stage (after 24 hours; right) of quorum sensing. Because *P. aeruginosa* is able to convert HHQ (**1**) to PQS, the PQS data measured after addition of **1** to the cultures represent a combination of auto-induction of AQ synthesis by HHQ, and biotransformation of HHQ to PQS. However, HHQ is not a biosynthetic precursor of HQNO. Means and standard errors of five biological replicates are shown. One-way ANOVA was used for statistical analysis, and significant differences compared to the control (C) are indicated (* *P* < 0.05, ** *P* < 0.01).

The compounds HHQ, PQS and HQNO are strongly related to quorum sensing and virulence in *P. aeruginosa*. HHQ and PQS act as signal molecules and, by interacting with the transcriptional regulator PqsR, induce the expression of the AQ biosynthetic genes. Such positive feedback or “auto-induction” loops are considered a hallmark of quorum sensing systems. Therefore, in order to detect potential effects of the methylated AQ derivatives on the AQ-based quorum sensing, we analyzed their influence on the production of PQS and HQNO by *P. aeruginosa* PAO1 (Figure 3).

For most compounds tested, PQS and HQNO contents of the cultures were unaffected compared to the DMSO control, indicating neither quorum sensing nor antiquorum sensing activity. However, besides the well-known quorum sensing signal molecule HHQ (**1**), also the two methylated PQS derivatives **11** and **12** and the HQNO derivative **6** elicited PQS and HQNO production by *P. aeruginosa* PAO1, suggesting quorum sensing activity. However, the significant activity of the HQNO derivative **6** is unexpected, as HQNO is not a signal. We assume that the weak N–O bond is cleaved under the assay conditions and HHQ is generated, which could be the cause of the observed effects. Experiments with a luciferase-based bioreporter strain [26] confirmed that compounds **11** and **12** act via addressing the transcriptional regulator PqsR of the AQ-based quorum sensing system, while compound **6** did not elicit a significant response (see Figure S2, Supporting Information File 1, for details). The data on the PQS derivatives **11** and **12** suggest that the presence of a substituent at one of the heteroatoms does not necessarily preclude quorum sensing activity, and that formation of a 1*H*-4-oxo-tautomeric form (which is impeded by the *O*-methylation in **11**) is not a prerequisite for quorum sensing activity [27]. Furthermore, in agreement with previous results, our results show the importance of the 3-hydroxy group, which turns non-active compounds **2** and **3** into PQS agonists, irrespective of 4-*O*-methylation (**11**) or *N*-methylation (**12**) [27–29].

## Conclusion

In this study we describe the synthesis of selectively methylated AQs and the influence of methylation on the bioactivity. While the methylation of HHQ is straightforward, selective preparation of methylated HQNO and PQS derivatives required optimized reaction conditions and multistep syntheses. The strategy to “oxidize” the 3-position of AQs via iodination, lithium–halogen exchange and electrophilic trapping can offer a flexible approach to new 3-substituted AQs starting from an easily available synthetic intermediate. While we decided to oxidize the newly formed boronic acid ester in situ, its isolation should be possible and enable further modification, for example by *ipso*-substitution with heteroatom electrophiles. The application of the boronic acid in Suzuki–Miyaura coupling should also be possible [30–31].

Growth inhibition experiments against *S. aureus* showed that HQNO derivatives (**5**, **6** and **7**) were generally more efficient than HHQ (**1**) and its derivatives (**2**–**4** and **8**). Interestingly, while PQS and 4OMe-PQS did not have any activity, NMe-PQS (**12**) was about as active as the HQNO derivatives. The 4-*O*-methylated derivatives of HQNO (**7**) and HHQ (**3**) but not the PQS derivative **11** were shown to reduce O_2_ consumption similar to HQNO, possibly indicating inhibition of the respiratory chain as proven for HQNO. However, the reduction in O_2_ consumption is less pronounced for HMOQ (**6**) and NMe-PQS (**12**). Therefore, it is possible that growth inhibition is following a different mechanism than for HQNO (**5**). The investigation of AQ levels in cultures of *P. aeruginosa* revealed quorum sensing activity for the two PQS derivatives **11** and **12**, suggesting once more that AQ-based quorum sensing in *P. aeruginosa* tolerates significant structural diversity on the signaling molecule [27–29].

## Experimental

Detailed synthetic procedures for all new compounds including copies of their NMR spectra can be found in Supporting Information File 1. HHQ and HQNO were prepared by adjusted and optimized literature procedures (see Supporting Information File 1 for details) [20–21]. 3-Iodo-2-heptyl-4(1*H*)-quinolone (3I-HHQ) was prepared according to a literature procedure [21].

### Bioassays

The growth of *S. aureus* strain Newman in the presence of AQs was followed by measuring the optical density of cell suspensions in 24-well microtiter plates. To this end, freshly grown cells were diluted in LB medium to an OD_600 nm_ of 0.2. Each well contained 1 mL of cell suspension including 100 µM of the AQ derivatives or an equal amount of DMSO. The plates were sealed with optically clear and gas permeable membranes, incubated at 37 °C while shaking and the OD_600 nm_ was measured at certain time points. Experiments were done in biological triplicates (*n* = 3).

The influence of AQs on the cellular consumption of O_2_ was studied as previously described [16]. Briefly, exponentially growing cells of *S. aureus* Newman were diluted in LB to an OD_600 nm_ of 0.5. After adding 100 µM AQs and incubating for 10 min, 500 µL cell suspension was transferred to the chamber of a Clark-type oxygen electrode and the O_2_ consumption was measured for 2–3 min. Experiments were done in biological triplicates (*n* = 3).

The effect of AQs on the quorum sensing activity in *P. aeruginosa* was studied by using AQ biosynthesis as measurable output. The induction of AQ biosynthesis by methylated AQs was followed by measuring PQS and HQNO concentrations in cultures of *P. aeruginosa* PAO1. Freshly grown cells were diluted to an OD_600 nm_ of 0.2 in LB and supplemented with 100 µM of AQ derivative. After shaking incubation for 4 hours and 24 hours at 37 °C, samples were taken and mixed with two volumes of acidified ethanol (2 g/L citric acid). After pelleting residual cells and debris by centrifugation, samples were analyzed by HPLC to determine PQS and HQNO concentrations [16].

## Supporting Information

File 1 Additional biological data, detailed synthetic procedures and copies of ^1^H and ^13^C NMR spectra of all compounds.
