# Supplementary material for: Synthesis and biological activity of methylated derivatives of the Pseudomonas metabolites HHQ, HQNO and PQS
Source: Beilstein J Org Chem. 2019 Jan 21;15:187–93. doi: 10.3762/bjoc.15.18 (PMC6350858; doi:10.3762/bjoc.15.18)
Supplement: File 1 — Additional biological data, detailed synthetic procedures and copies of 1H and 13C NMR spectra of all compounds. [file Beilstein_J_Org_Chem-15-187-s001.pdf]

BEILSTEIN JOURNAL OF ORGANIC CHEMISTRY

Supporting Information

for

Synthesis and biological activity of methylated derivatives of the *Pseudomonas* metabolites HHQ, HQNO and PQS

Sven Thierbach, Max Wienhold, Susanne Fetzner and Ulrich Hennecke

Beilstein J. Org. Chem. **2019,** *15,* 187–193. doi:10.3762/bjoc.15.18

Additional biological data, detailed synthetic procedures and copies of ^{1}H and ^{13}C NMR spectra of all compounds

License and Terms: This is a supporting information file under the terms of the Creative Commons Attribution License (http://creativecommons.org/licenses/by/4.0). Please note that the reuse, redistribution and reproduction in particular requires that the authors and source are credited.
The license is subject to the *Beilstein Journal of Organic Chemistry* terms and conditions: (https://www.beilstein-journals.org/bjoc)

Contents

1. Assay on the formation of static biofilms S2
2. PqsR bioreporter assay S3
3. Chemical synthesis S4
4. NMR spectra S12
5. Literature S25

1. Assay on the formation of static biofilms

To evaluate the influence of AQ derivatives on the formation of static biofilms, the method of O'Toole was slightly modified [1]. In brief, cultures of *S. aureus* Newman were adjusted to an $OD_{600\ nm}$ of 0.1 in LB and 100 µL were used to inoculate 96-well microtiter plates. After incubation for 24 hours at 37 °C without shaking, the biofilm was stained with 0.1 % (w/v) crystal violet and the absorbance at 560 nm (A560 nm) was measured. Prior to staining, OD_{600} nm was measured, indicating the total biomass. By normalizing the biofilm to the total biomass, effects of AQs on biofilm formation rather than growth are indicated. Analyses included six biological replicates (n = 6), each measured in quadruplicate.

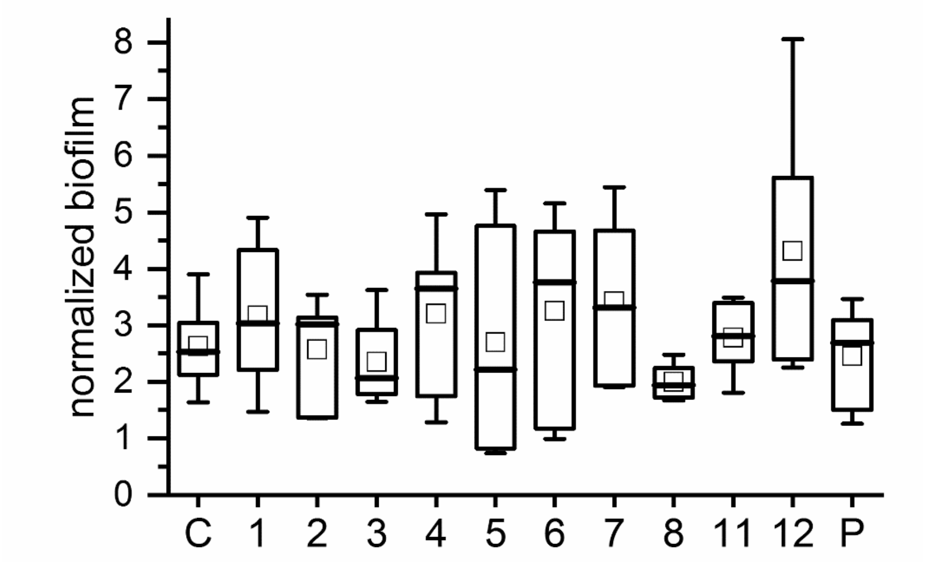

Figure S1: Effect of AQs on biofilm formation by *S. aureus*. Cell suspensions were incubated for 24 h with 100 µM of the indicated AQ (**1**–**12**), PQS (P) or an equal amount of DMSO as the control (C). The total biofilm mass, as measured by crystal violet staining, was normalized to the cell density measured prior to biofilm staining. Box-plot shows the medians (center lines), the means (squares), interquartile ranges (box), and 1.5 × interquartile ranges (whiskers) of 6 biological replicates (n = 6) with 4 technical repeats per replicate. The Mann–Whitney test was used for statistical analysis, but no significant differences ($P < 0.05$) were found.

2. PqsR bioreporter assay

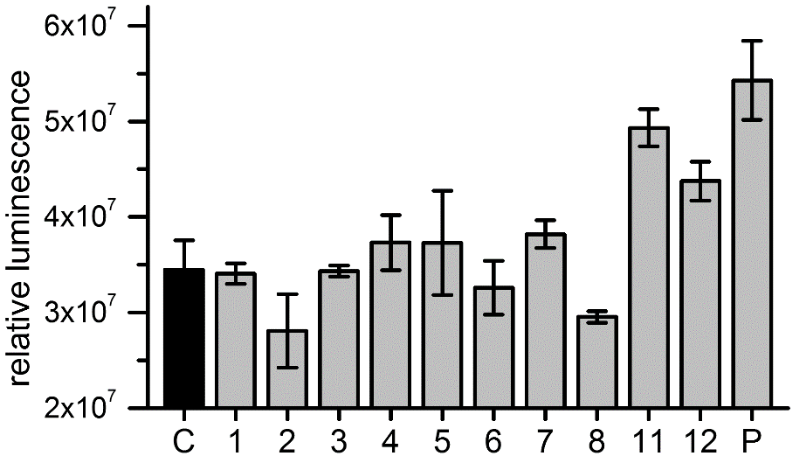

Figure S2: Competition of AQ derivatives with PQS for the binding to PqsR, the transcriptional regulator of the AQ biosynthesis gene cluster. The bioreporter strain *Pseudomonas putida* KT2440 mini-Tn*7*T::*pqsR*-P_{pqsA}-*lux* harbors the gene *pqsR* (constitutively expressed) for the transcriptional regulator PqsR, which upon binding of PQS (or HHQ) upregulates the expression of the AQ biosynthesis gene cluster, and the luciferase genes *luxCDABE* under control of the PqsR-responsive *pqsA* promoter [2]. The bioreporter was used to verify that the newly synthesized AQ derivatives have direct impact on the PqsR-dependent transcription and to identify agonistic or antagonistic effects. To this end, cultures of the bioreporter strain ($OD_{600\ nm}$ of 0.2 in LB) were supplemented with 0.5 µM of PQS, and additionally with 10 µM of the indicated AQ (**1**–**12**), PQS (P), or an equal amount of DMSO (C). After 4 h of incubation at 20 °C, Lux-based bioluminescence was measured and normalized to the $OD_{600\ nm}$ of the culture (relative luminescence). Bars and errors represent the means and standard errors of biological triplicates.

3. Chemical synthesis

General information

Manipulations involving air- or moisture-sensitive reagents were carried out under argon and performed by using standard *Schlenk*-techniques. Dry and oxygen-free solvents were employed for such reactions. Solvents for extraction and flash chromatography were distilled before use. Commercial compounds were purchased from standard suppliers (ABCR, Acros, Alfa Aesar, Sigma-Aldrich or TCI) and used without further purification. Flash chromatography (FC) was carried out on Acros silica gel (0.035–0.070 mm; 60 Å) under slight overpressure. IR spectra were recorded with a Digilab FTS 4000 with an attenuated total reflectance (ATR) unit. NMR spectroscopic data were recorded on a Bruker DPX-300 (300 MHz), Bruker AV400 (400 MHz) or Agilent DD2 600 (600 MHz). Chemical shifts (δ) are reported in ppm relative to $SiMe_4$ (^1H, ^{13}C) and referenced to either the residual solvent signal for ^1H NMR or the solvent signal for ^{13}C NMR (δ = 7.26/77.2 ppm for $CDCl_3$; δ = 2.50/39.5 ppm for DMSO-d_6). NMR multiplicities are abbreviated as follows: s = singlet, d = doublet, t = triplet, sept = septet, m = multiplet, br = broad signal. Mass spectra with electrospray ionization (MS-ESI-EM, *m*/*z*) were recorded on a Bruker MicroTof or a Thermo Scientific Orbitrap LTQ XL (Nanospray).

Synthetic procedures

Methyl 3-oxodecanoate [3,4]

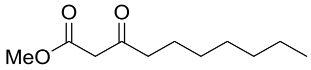

Meldrum's acid (28.8 g, 200 mmol, 1.0 equiv) was dissolved in dry CH_2Cl_2 (200 mL) and pyridine (32 mL, 400 mmol, 2.0 equiv) and DMAP (488 mg, 4 mmol, 0.02 equiv) was added. The solution was cooled to 0 °C and octanoyl chloride (37.6 mL, 220 mmol, 1.1 equiv) was added dropwise at this temperature. The solution was allowed to warm to rt and stirred for 2 h. Aqueous HCl was added (2 M, 200 mL) and the phases were separated. The organic phase was washed with aqueous HCl (2 M, 2 × 200 mL) and water (2 × 200 mL), dried over sodium sulfate and the solvent was removed in vacuo. The crude product was dissolved in MeOH (160 mL) and heated to 65 °C for 16 h. The solvent was removed in vacuo and the residue was dissolved in EtOAc (200 mL). The solution was washed with aqueous K_2CO_3 (10% in H_2O, 2*80 mL) and water (80 mL), dried over sodium sulfate and the solvent was removed in vacuo. The residue was distilled under vacuum using a Vigreux-column to give the product (bp 75 °C at 0.01 Torr, 20.3 g, 101 mmol, 51%) as a colourless oil.

Found: C, 65.8; H, 9.9. Calc. for $C_{11}H_{20}O_3$: C, 66.0; H, 10.1%. IR (film): ṽ (cm^{-1}) = 2955*w*, 2928*m*, 2856*m*, 1747*s*, 1716*s*, 1656*w*, 1629*w*, 1437*m*, 1407*w*, 1318*m*, 1235*m*, 1152*m*, 1077*m*, 1003*w*. ^{1}H-NMR (400 MHz, $CDCl_3$, 296 K): δ = 3.72 (s, 3H), 3.43 (s, 2H), 2.51 (t, 3H, ^3J = 7.4 Hz), 1.62-1.52 (m, 2H), 1.33-1.19 (m, 8H), 0.86 (t, 3H, ^3J = 6.7 Hz). ^{13}C-NMR (100 MHz, $CDCl_3$, 296 K): δ = 203.0, 167.8, 52.4, 49.1, 43.2, 31.7, 29.1, 29.1, 23.6, 22.7, 14.2. MS-ESI-EM: m/z = 223.1305 calculated for $C_{11}H_{20}O_3Na$ $[M+Na]^+$, found: 223.1310. NMR data are given for the main tautomer (about 10% of the enol tautomer are present).

2-Heptyl-4(1*H*)-quinolone (HHQ, 1) [3,4]

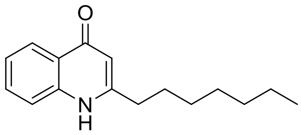

Methyl 3-oxodecanoate (20.3 g, 101 mmol, 1.00 equiv) was dissolved in toluene (100 mL). Aniline (9.7 mL, 106 mmol, 1.05 equiv) and *p*-toluenesulfonic acid monohydrate (386 mg, 2 mmol, 0.02 equiv) were added and the solution was heated to 110 °C for 3 h (until no water was formed anymore) using a Dean–Stark apparatus. After cooling to rt the solvent was removed in vacuo (removal of traces of toluene under high vacuum is recommended to facilitate the next step) to provide a yellowish oil. In a two-neck round-bottom flask equipped with a condenser and a dropping funnel diphenyl ether (25 mL) was heated to boiling temperature (258 °C). The yellowish oil from the first step was dissolved in diphenyl ether (10 mL) and added dropwise to the refluxing diphenyl ether via the dropping funnel over a period of 30 min. The resulting solution was heated to 258 °C for another 30 min. The solution was allowed to cool to rt and heptane (70 mL) was added when the solution had cooled to about 50 °C. The solution was allowed to stand at rt for 14 h. The white precipitate was filtered off, washed with heptane (35 mL) and suspended in heptane (70 mL). The suspension was heated to 98 °C for 1 h, filtered and the white solid was collected and dried under vacuum to provide the product (10.7 g, 44 mmol, 44%).

M.p. 145 °C (heptane/Ph_2O). Found: C, 78.8; H, 8.65; N, 5.8. Calc. for $C_{16}H_{21}NO$: C, 79.0; H, 8.7; N, 5.8%. IR (film): ṽ (cm^{-1}) = 3061*w*, 2922*m*, 2849*m*, 1637*m*, 1593*s*, 1550*s*, 1501*s*, 1472*s*, 1438*s*, 1355*m*, 1321*m*, 1246*w*, 1169*w*, 1137*m*, 1111*w*, 837*m*, 779*m*, 751*s*. ^{1}H-NMR (300 MHz, $CDCl_3$, 300 K): δ = 12.47 (br s, 1H), 8.40-8.33 (m, 1H), 7.85-7.77 (m, 1H), 7.62-7.54 (m, 1H), 7.37-7.28 (m, 1H), 6.25 (s, 1H), 2.76-2.64 (m, 2H), 1.79-1.63 (m, 2H), 1.34-1.08 (m, 8H), 0.81 (t, 3H, ^3J = 6.8 Hz). ^{13}C-NMR (75 MHz, $CDCl_3$, 300 K): δ = 179.0, 155.6, 140.9, 131.9, 125.4, 125.1, 123.7, 118.8, 108.2, 34.5, 31.8, 29.3, 29.3, 29.1, 22.7, 14.1. MS-ESI-EM: m/z = 244.1696 calculated for $C_{16}H_{21}NOH$ $[M+H]^+$, found: 244.1705.

N-Methyl-2-heptyl-4(1H)-quinolone (NMe-HHQ, 2)

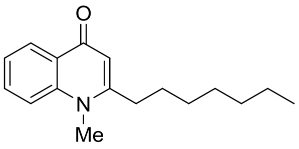

HHQ (**1**, 243 mg, 1.0 mmol, 1.0 equiv) was dissolved in THF (10 mL) and the solution was cooled to 0 °C. *n*-Butyllithium (1.6 M in hexane, 0.7 mL, 1.1 mmol, 1.1 equiv) was added dropwise and the solution was stirred at 0 °C for 45 min. Dimethyl sulfate (0.12 mL, 1.3 mmol, 1.3 equiv) was added and the solution was allowed to warm to rt and stirred at rt for 14 h. Concentrated aqueous ammonia (10 mL) was added and the solution was stirred for 15 min. EtOAc (10 mL) was added, the phases were separated and the aqueous phase was extracted with EtOAc (10 mL). The organic phases were combined, washed with water (10 mL), dried over $MgSO_4$ and the solvent was removed in vacuo. The crude product was purified by column chromatography (CH_2Cl_2/MeOH = 25:1) to give **2** (131 mg, 0.51 mmol, 51%) as a white solid.

IR (film): ṽ (cm^{-1}) = 2925*m*, 2856*w*, 1629*s*, 1597*s*, 1572*m*, 1552*m*, 1498*m*, 1467*m*, 1175*w*, 844*w*. ^{1}H-NMR (300 MHz, $CDCl_3$, 300 K): δ = 8.48-8.40 (m, 1H); 7.68-7.60 (m, 1H); 7.53-7.45 (m, 1H); 7.39-7.31 (m, 1H); 6.22 (s, 1H); 3.72 (s, 3H); 2.75-2.64 (m, 2H); 1.75-1.61 (m, 2H); 1.49-1.21 (m, 8H); 0.88 (t, 3H, ^3J = 6.9 Hz). ^{13}C-NMR (75 MHz, $CDCl_3$, 300 K): δ = 177.9, 154.8, 142.1, 132.1, 126.8, 126.8, 123.4, 115.3, 111.3, 34.9, 34.2, 31.8, 29.4, 29.1, 28.7, 22.7, 14.2. MS-ESI-EM: m/z = 258.1852 calculated for $C_{17}H_{23}NOH$ $[M+H]^+$, found: 258.1850.

4-Methoxy-2-heptylquinoline (OMe-HHQ, 3) and N-methyl-2-(1-methylheptyl)-4(1H)-quinolone (4)

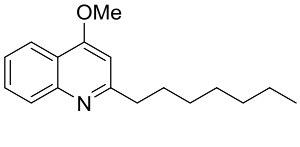

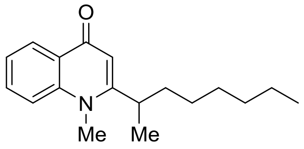

In a *Schlenk*-type pressure tube HHQ (**1**, 356 mg, 1.5 mmol, 1.0 equiv) was dissolved in acetone (10 mL). K_2CO_3 (622 mg, 4.5 mmol, 3.0 equiv) and methyl iodide (0.28 mL, 4.5 mmol, 3.0 equiv) were added and the resulting suspension was stirred at 80 °C for 4 h. The mixture was allowed to cool to rt and the solvent was removed in vacuo. The residue was dissolved in CH_2Cl_2 (25 mL), washed with water (20 mL), dried over $MgSO_4$ and the solvent was removed

in vacuo. The crude product was purified by column chromatography (pentane/EtOAc = 2:1, followed by EtOAc) to give **3** (122 mg, 0.47 mmol, 32%) as a colourless oil and **4** (55 mg, 0.20 mmol, 14%) as a colourless oil.

4-Methoxy-2-heptylquinoline (**3**)

IR (film): ṽ (cm^{-1}) = 2926*m*, 2853*w*, 1620*w*, 1596*s*, 1565*m*, 1507*m*, 1457*w*, 1420*m*, 1234*w*, 1196*w*, 1159*w*, 1114*m*, 993*w*, 764*s*, 631*s*. ^{1}H-NMR (300 MHz, $CDCl_3$, 300 K): δ = 8.16-8.11 (m, 1H), 7.99-7.94 (m, 1H), 7.68-7.61 (m, 1H), 7.46-7.40 (m, 1H), 6.63 (s, 1H), 4.04 (s, 3H), 2.96-2.87 (m, 2H), 1.87-1.74 (m, 2H), 1.49-1.21 (m, 8H), 0.88 (t, 3H, ^3J = 6.9 Hz). ^{13}C-NMR (75 MHz, $CDCl_3$, 300 K): δ = 164.5, 162.5, 148.9, 129.8, 128.4, 124.9, 121.7, 120.2, 99.9, 55.7, 40.2, 31.9, 30.3, 29.8, 29.4, 22.8, 14.2. ESI-MS-EM: m/z = 258.1852 calculated for $C_{17}H_{23}NOH$ $[M+H]^+$, found: 258.1851.

N-Methyl-2-(1-methylheptyl)-4(1*H*)-quinolone (**4**)

IR (film): ṽ (cm^{-1}) = 2927*m*, 2857*w*, 1622*s*, 1598*s*, 1572*m*, 1552*m*, 1498*m*, 1469*s*, 842*w*, 759*m*. ^{1}H-NMR (300 MHz, $CDCl_3$, 300 K): δ = 8.44-8.36 (m, 1H); 7.65-7.57 (m, 1H); 7.53-7.46 (m, 1H); 7.35-7.27 (m, 1H); 6.28 (s, 1H); 3.74 (s, 3H); 3.03-2.90 (m, 1H); 1.75-1.46 (m, 2H); 1.38-1.15 (m, 11H); 0.83 (t, 3H, ^3J = 6.9 Hz). ^{13}C-NMR (75 MHz, $CDCl_3$, 300 K): δ = 178.0, 159.9, 142.2, 132.0, 126.5, 126.5, 123.3, 115.8, 108.3, 36.5, 35.9, 33.9, 31.7, 29.3, 27.4, 22.6, 20.4, 14.1. MS-ESI-EM: m/z = 272.2009 calculated for $C_{18}H_{25}NOH$ $[M+H]^+$, found: 272.2013.

***N*-Hydroxy-2-heptyl-4(1*H*)-quinolone (HQNO) [5]**

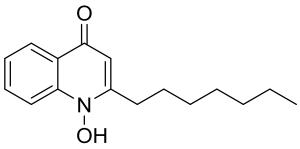

HHQ (1944 mg, 8.0 mmol, 1.0 equiv) was dissolved in THF (40 mL) and KO*t*-Bu (1120 mg, 10.0 mmol, 1.25 equiv) was added at rt. The solution was stirred at rt for 30 min, Boc_2O (1918 mg, 8.8 mmol, 1.1 equiv) was added and the solution was stirred at rt for 14 h. The reaction mixture was diluted with EtOAc (120 mL), washed with water (100 mL), dried over $MgSO_4$ and the solvent was removed in vacuo. The crude product was dissolved in CH_2Cl_2 (40 mL), cooled to 0 °C and *m*CPBA (wt.75%, 2024 mg, 8.8 mmol, 1.1 equiv) was added at this temperature. The solution was stirred for 4 h at 0 °C. CH_2Cl_2 (40 mL) was added, the solution was washed with aqueous, saturated Na_2CO_3 solution (2 × 60 mL) and water (60 mL), dried over $MgSO_4$ and the solvent was removed in vacuo. The crude product was dissolved in EtOH (40 mL) and aqueous KOH-solution (5 M, 20 mL) was added at rt. The solution was stirred at rt for 1 h. Water (120 mL) was added and the pH of the solution was adjusted to about pH 2 using concentrated aqueous HCl. The resulting white precipitate was filtered off and crystallized from EtOH/water (4:1) to give the product (1432 mg, 5.5 mmol, 69%) as a crystalline white solid.

M.p. 160-161 °C (EtOAc). IR (film): ṽ (cm^{-1}) = 2923*m*, 2854*m*, 1590*s*, 1547*s*, 1453*s*, 1177*s*, 755*s*, 719*m*. ^{1}H-NMR (300 MHz, $CDCl_3$, 300 K): δ = 14.17 (br s, 1H), 8.29-8.21 (m, 1H), 8.12-8.05 (m, 1H), 7.62-7.54 (m, 1H), 7.40-7.32 (m, 1H), 6.26 (s, 1H), 2.72-2.61 (m, 2H), 1.58-1.44 (m, 2H), 1.30-1.12 (m, 8H), 0.84 (t, 3H, ^3J = 6.7 Hz). ^{13}C-NMR (75 MHz, $CDCl_3$, 300 K): δ = 170.6, 155.7, 140.7, 132.3, 125.0, 124.9,

123.8, 116.7, 105.6, 31.9, 31.7, 29.6, 29.1, 27.5, 22.7, 14.2. MS-ESI-EM: m/z = 260.1645 calculated for $C_{16}H_{21}NO_2H$ $[M+H]^+$, found: 260.1642.

3-Iodo-2-heptyl-4(1*H*)-quinolone (3I-HHQ) [4]

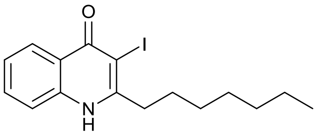

2-Heptyl-4(1*H*)-quinolone (333 mg, 1.37 mmol, 1.0 equiv) was dissolved in AcOH (10 mL) at rt. NIS (315 mg, 1.40 mmol, 1.0 equiv) was added in one portion and the solution was stirred for 3 h at rt. The precipitate was collected by filtration, washed with AcOH (10 mL) and MeCN (6 mL) and crystallized from EtOH to give the product (241 mg, 0.65 mmol, 48 %) as colourless crystalline solid.

M.p. 224-225 °C (EtOH). IR (film): ṽ (cm^{-1}) = 3257*w*, 3210*w*, 3060*w*, 2923*s*, 2851*m*, 1627*s*, 1577*m*, 1554*s*, 1496*m*, 1473*s*, 1436*m*, 1378*w*, 1351*m*, 1135*m*, 1013*m*, 754*s*, 685*s*, 626*m*, 569*m*. ^{1}H-NMR (400 MHz, DMSO-d_6, 300 K): δ = 12.05 (br s, 1H), 8.09-8.04 (m, 1H), 7.70-7.64 (m, 1H), 7.60-7.54 (m, 1H), 7.38-7.31 (m, 1H), 2.94-2.86 (m, 2H), 1.73-1.62 (m, 2H), 1.44-1.21 (m, 8H), 0.85 (t, 3H, ^3J = 6.8 Hz). ^{13}C-NMR (100 MHz, DMSO-d_6, 300 K): δ = 173.2, 154.5, 139.0, 132.0, 125.5, 123.8, 120.6, 117.8, 85.8, 38.7, 31.1, 28.7, 28.3, 28.0, 22.1, 14.0. MS-ESI-EM: m/z = 392.0482 calculated for $C_{16}H_{20}INONa$ $[M+Na]^+$, found: 392.0482.

***N*-Methoxy-2-heptyl-4(1*H*)-quinolone (HMOQ, 6) [6]**

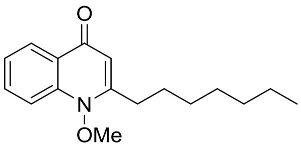

HQNO (**5**) (83 mg, 0.32 mmol) was dissolved in methanol (5 mL) and diethyl ether (2 mL). The solution was cooled to 0 °C and trimethylsilyldiazomethane (2 M solution in diethyl ether; 0.32 mL, 0.64 mmol, 2.0 equiv) was added dropwise. The solution was stirred for 1 h at 0 °C before another portion of trimethylsilyldiazomethane (2 M solution in diethyl ether; 0.32 mL, 0.64 mmol, 2.0 equiv.) was added dropwise and the solution was stirred for another 1 h at 0 °C. Formic acid (0.5 mL) was added and the solution was stirred for 15 min at 0 °C. Ethyl acetate (15 mL) and sodium hydrogencarbonate solution (aqueous, sat., 15 mL) were added, the phases were separated and the aqueous phase was extracted with ethyl acetate (15 mL). The organic phases were combined, dried over magnesium sulfate and the solvent was removed in vacuo. The residue was purified by column chromatography (silica gel, ethyl acetate) to provide the product **6** (20 mg, 0.07 mmol, 23%) as a colourless oil.

IR (film): ṽ (cm^{-1}) = 2926*m*, 2855*w*, 1625*s*, 1598*s*, 1574*m*, 1555*m*, 1487*m*, 1461*m*, 1427*m*, 1337*w*, 1113*w*, 968*w*, 777*w*, 753*m*. ^{1}H-NMR (600 MHz, $CDCl_3$, 298 K): δ = 8.38-8.36 (m, 1H); 7.70-7.66 (m, 1H); 7.61-7.58 (m, 1H); 7.38-7.35 (m, 1H); 6.10 (s, 1H); 4.02 (s, 3H); 2.91-2.83 (m, 1H); 2.66-2.57 (m, 1H); 1.83-1.66 (m, 2H); 1.46-1.38 (m, 2H); 1.37-1.24 (m, 6H); 0.88 (t, 3H, ^3J = 7.2 Hz). ^{13}C-NMR (151 MHz, $CDCl_3$, 298 K): δ = 177.2, 152.7, 138.3, 132.5, 126.9,

125.9, 123.9, 113.5, 108.8, 64.5, 31.8, 30.6, 29.3, 29.1, 28.2, 22.7, 14.2. MS-ESI-EM: m/z = 274.1802 calculated for $C_{17}H_{23}NO_2H$ $[M+H]^+$, found: 274.1801.

4-Methoxy-2-heptylquinoline *N*-oxide (4OMe-HQNO, 7)

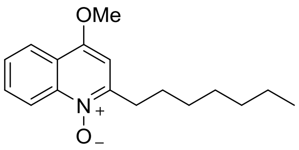

4-Methoxy-2-heptylquinoline (**3**) (220 mg, 0.86 mmol, 1.0 equiv) was dissolved in CH_2Cl_2 (10 mL) and *m*CPBA (75 *wt*%, 236 mg, 1.03 mmol, 1.2 equiv) was added at rt. The solution was stirred at rt for 14 h. Aqueous Na_2CO_3-solution (1 M, 10 mL) was added, the phases were separated and the aqueous phase was extracted with CH_2Cl_2 (10 mL). The organic phases were combined, washed with aqueous Na_2CO_3 solution (1 M, 10 mL), dried over $MgSO_4$ and the solvent was removed in vacuo. The crude product was purified by column chromatography (EtOAc) to give **7** (106 mg, 0.39 mmol, 45%) as a colourless oil.

IR (film): ṽ (cm^{-1}) = 2924*m*, 2855*m*, 1606*s*, 1568*m*, 1451*m*, 1375*s*, 1211*s*, 1122*s*, 1092*s*, 978*m*, 768*s*, 734*m*. ^{1}H-NMR (400 MHz, $CDCl_3$, 296 K): δ = 8.80-8.72 (m, 1H), 8.18-8.10 (m, 1H), 7.81-7.71 (m, 1H), 7.60-7.51 (m, 1H), 6.59 (s, 1H), 4.03 (s, 3H), 3.17-3.07 (m, 2H), 1.88-1.75 (m, 2H), 1.52-1.18 (m, 8H), 0.87 (t, 3H, ^3J = 6.9 Hz). ^{13}C-NMR (101 MHz, $CDCl_3$, 296 K): δ = 153.8, 150.2, 141.5, 131.0, 127.1, 122.4, 121.6, 119.9, 100.5, 56.1, 32.4, 31.9, 29.8, 29.2, 26.5, 22.8, 14.2. ESI-MS-EM: m/z = 296.1621 calculated for $C_{17}H_{23}NO_2Na$ $[M+Na]^+$, found: 296.1621.

3-Iodo-4-methoxy-2-heptylquinoline (9) and 3-iodo-*N*-methyl-2-heptyl-4(1*H*)-quinolone (10)

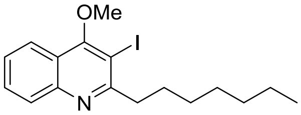

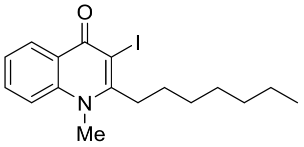

In a *Schlenk*-type pressure tube 2-heptyl-3-iodo-4(1*H*)quinolone (**8**) (241 mg, 0.65 mmol, 1.0 equiv) was dissolved in acetone (5 mL). K_2CO_3 (271 mg, 1.96 mmol, 3.0 equiv) and methyl iodide (0.12 mL, 1.96 mmol, 3.0 equiv) were added and the resulting suspension was stirred at 80 °C for 4 h. The mixture was allowed to cool to rt and the solvent was removed in vacuo. The residue was dissolved in CH_2Cl_2 (25 mL), washed with water (20 mL), dried over $MgSO_4$ and the solvent was removed in vacuo. The crude product was purified by column chromatography (pentane/EtOAc = 3:1) to give **9** (52 mg,0.14 mmol, 21%) as a colourless oil and **10** (60 mg, 0.16 mmol, 24%) as a colourless oil.

3-Iodo-4-methoxy-2-heptylquinoline (**9**)

IR (film): ṽ (cm^{-1}) = 2924*s*, 2853*m*, 1571*s*, 1488*s*, 1455*w*, 1367*s*, 1082*m*, 1003*m*, 765*s*. ^{1}H-NMR (300 MHz, $CDCl_3$, 300 K): δ = 8.07-8.00 (m, 2H), 7.74-7.67 (m, 1H), 7.54-7.47 (m, 1H), 4.04 (s, 3H), 3.24-3.15 (m, 2H), 1.88-1.74 (m, 2H), 1.56-1.23 (m, 8H), 0.89 (t, 3H, ^3J = 6.9 Hz). ^{13}C-NMR (75 MHz, $CDCl_3$, 300 K): δ = 164.6, 164.2, 149.2, 130.3, 129.1, 126.4, 122.5, 122.0, 89.6, 61.9, 42.6, 32.0, 29.9, 29.7, 29.3, 22.8, 14.3. MS-ESI-EM: m/z = 384.0819 calculated for $C_{17}H_{22}INOH$ $[M+H]^+$, found: 384.0822.

3-Iodo-*N*-methyl-2-heptyl-4(1*H*)-quinolone (**10**)

IR (film): ṽ (cm^{-1}) = 2924*m*, 2853*m*, 1615*s*, 1589*s*, 1518*s*, 1460*s*, 1396*m*, 1345*w*, 1262*m*, 1179*m*, 1075*m*, 912*w*, 756*s*, 687*m*, 649*w*. ^{1}H-NMR (300 MHz, $CDCl_3$, 300 K): δ = 8.34 (dd, ^{3}J = 8.0, 1.7 Hz, 1H), 7.61-7.54 (m, 1H), 7.46-7.40 (m, 1H), 7.30-7.23 (m, 1H), 3.81 (s, 3H), 3.17-3.07 (m, 2H), 1.68-1.54 (m, 2H), 1.53-1.23 (m, 8H), 0.88 (t, 3H, ^{3}J = 6.9 Hz). ^{13}C-NMR (75 MHz, $CDCl_3$, 300 K): δ = 173.7, 155.1, 140.8, 132.3, 127.6, 124.1, 122.5, 115.4, 90.4, 40.1, 36.7, 31.7, 29.6, 28.9, 27.5, 22.6, 14.1. MS-ESI-EM: m/z = 406.0638 calculated for $C_{17}H_{22}INONa$ [M+Na]$^{+}$, found: 406.0647.

3-Hydroxy-4-methoxy-2-heptylquinoline (4OMe-PQS, 11).

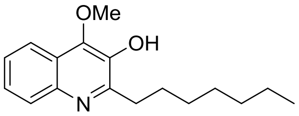

9 (36 mg, 0.09 mmol, 1 equiv) was dissolved in dry THF (2 mL) and the solution was cooled to −78 °C. *n*-Butyllithium (1.6 M in hexane, 70 µL, 0.11 mmol, 1.2 equiv) was added dropwise and the solution was stirred at −78 °C for 30 min. Trimethyl borate (16 µL, 0.14 mmol, 1.5 equiv) was added and the solution was allowed to warm to rt and stirred for 14 h. Sodium perborate tetrahydrate (72 mg, 0.47 mmol, 5.0 equiv) and water (2 mL) were added and the mixture was stirred at rt for 5 h. EtOAc (10 mL) and aqueous, saturated NH_4Cl solution (10 mL) were added, the phases were separated and the aqueous phase was extracted with EtOAc (10 mL). The organic phases were combined, washed with brine (10 mL), dried over $MgSO_4$ and the solvent was removed *in vacuo*. The crude product was purified by column chromatography (pentane/EtOAc = 9:1) to give **11** (9 mg, 0.03 mmol, 35%) as a colourless oil.

IR (film): ṽ (cm^{-1}) = 2927*m*, 2854*m*, 1599*m*, 1501*m*, 1465*w*, 1378*s*, 1280*w*, 1219*w*, 1069*m*, 971*w*, 762*s*. ^{1}H-NMR (300 MHz, $CDCl_3$, 300 K): δ = 8.05-7.99 (m, 2H), 7.91-7.86 (m, 1H), 7.56-7.43 (m, 2H), 6.27 (br s, 1H), 4.02 (s, 3H), 3.08-2.98 (m, 2H), 1.87-1.74 (m, 2H), 1.49-1.20 (m, 8H), 0.87 (t, 3H, ^{3}J = 6.9 Hz). ^{13}C-NMR (75 MHz, $CDCl_3$, 300 K): δ = 156.1, 145.8, 144.1, 140.3, 129.3, 126.8, 125.9, 122.7, 120.4, 61.9, 34.1, 32.0, 29.9, 29.3, 28.6, 22.8, 14.2. MS-ESI-EM: m/z = 274.1802 calculated for $C_{17}H_{23}NO_2H$ [M+H]$^{+}$, found: 274.1814.

3-Hydroxy-*N*-methyl-2-heptyl-4(1*H*)-quinolone (NMe-PQS, 12)

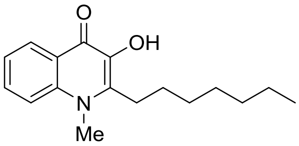

10 (308 mg, 0.80 mmol, 1 equiv) was dissolved in dry THF (10 mL) and the solution was cooled to −78 °C. *n*-Butyllithium (1.6 M in hexane, 0.6 mL, 0.96 mmol, 1.2 equiv) was added dropwise and the solution was stirred at −78 °C for 30 min. Trimethyl borate (0.13 mL, 1.20 mmol, 1.5 equiv) was added and the solution was allowed to warm to rt and stirred for 14 h. Sodium perborate tetrahydrate (619 mg, 4.00 mmol, 5.0 equiv) and water (10 mL) were added and the mixture was stirred at rt for 5 h. EtOAc (20 mL) and aqueous, saturated NH_4Cl-solution (20 mL) were added, the phases were separated and the aqueous phase was extracted with

EtOAc (20 mL). The organic phases were combined, washed with brine (20 mL), dried over $MgSO_4$ and the solvent was removed in vacuo. The crude product was purified by column chromatography (pentane/EtOAc = 2:1) to give **12** (30 mg, 0.11 mmol, 14%) as a colourless oil.

IR (film): $\tilde{\nu}$ (cm^{-1}) = 2927*m*, 2857*m*, 1620*m*, 1577*s*, 1541*s*, 1310*s*, 1244*m*, 1128*w*, 1098*w*, 755m. ^{1}H-NMR (300 MHz, $CDCl_3$, 300 K): δ = 8.51-8.44 (m, 1H), 7.67-7.59 (m, 1H), 7.57-7.51 (m, 1H), 7.39-7.231 (m, 1H), 3.84 (s, 3H), 3.07-2.97 (m, 2H), 1.74-1.60 (m, 2H), 1.53-1.21 (m, 8H), 0.89 (t, 3H, ^3J = 6.9 Hz). ^{13}C-NMR (75 MHz, $CDCl_3$, 300 K): δ = 169.0, 139.3, 139.3, 137.3, 131.3, 126.4, 123.0, 122.6, 115.5, 34.6, 31.9, 29.6, 29.2, 28.4, 27.5, 22.8, 14.2. MS-ESI-EM: m/z = 296.1621 calculated for $C_{17}H_{23}NO_2Na$ $[M+Na]^+$, found: 296.1607.

4. NMR spectra

3-Oxodecanoic acid methylester

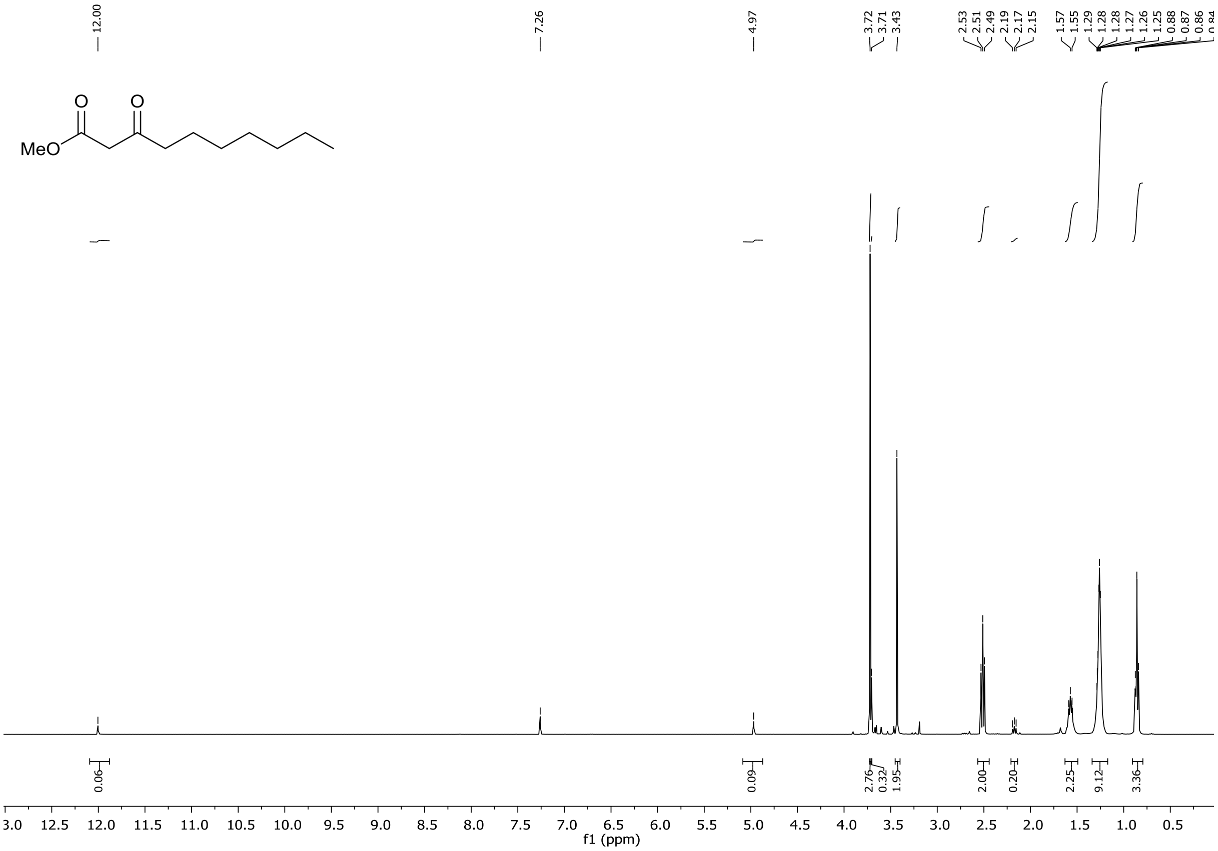

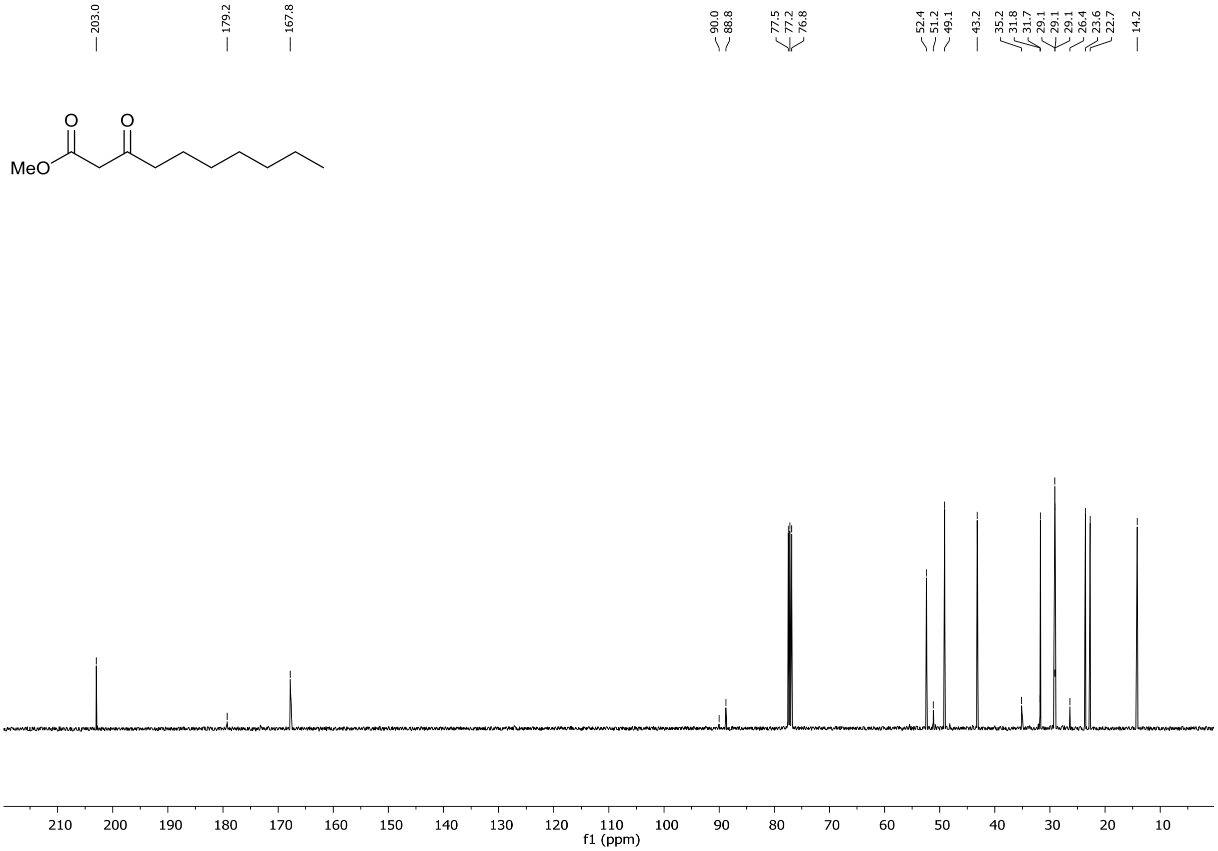

HHQ (1)

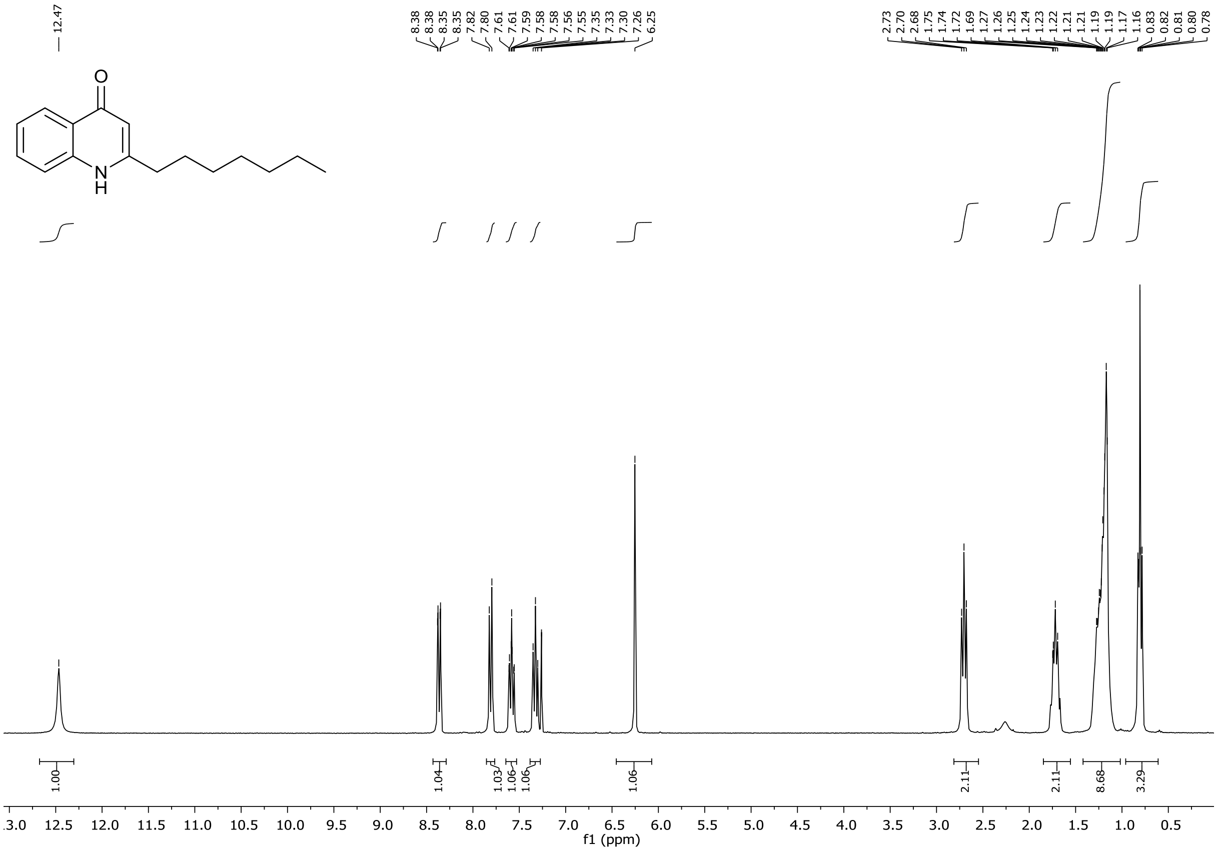

12.47
8.38
8.38
8.35
8.35
7.82
7.80
7.61
7.61
7.59
7.58
7.58
7.56
7.55
7.35
7.33
7.30
7.26
6.25
2.73
2.70
2.68
1.75
1.74
1.72
1.69
1.27
1.26
1.25
1.24
1.23
1.22
1.21
1.21
1.19
1.19
1.17
1.16
0.83
0.82
0.81
0.80
0.78
O
N
H
1.00
1.04
1.03
1.06
1.06
1.06
2.11
2.11
8.68
3.29
f1 (ppm)

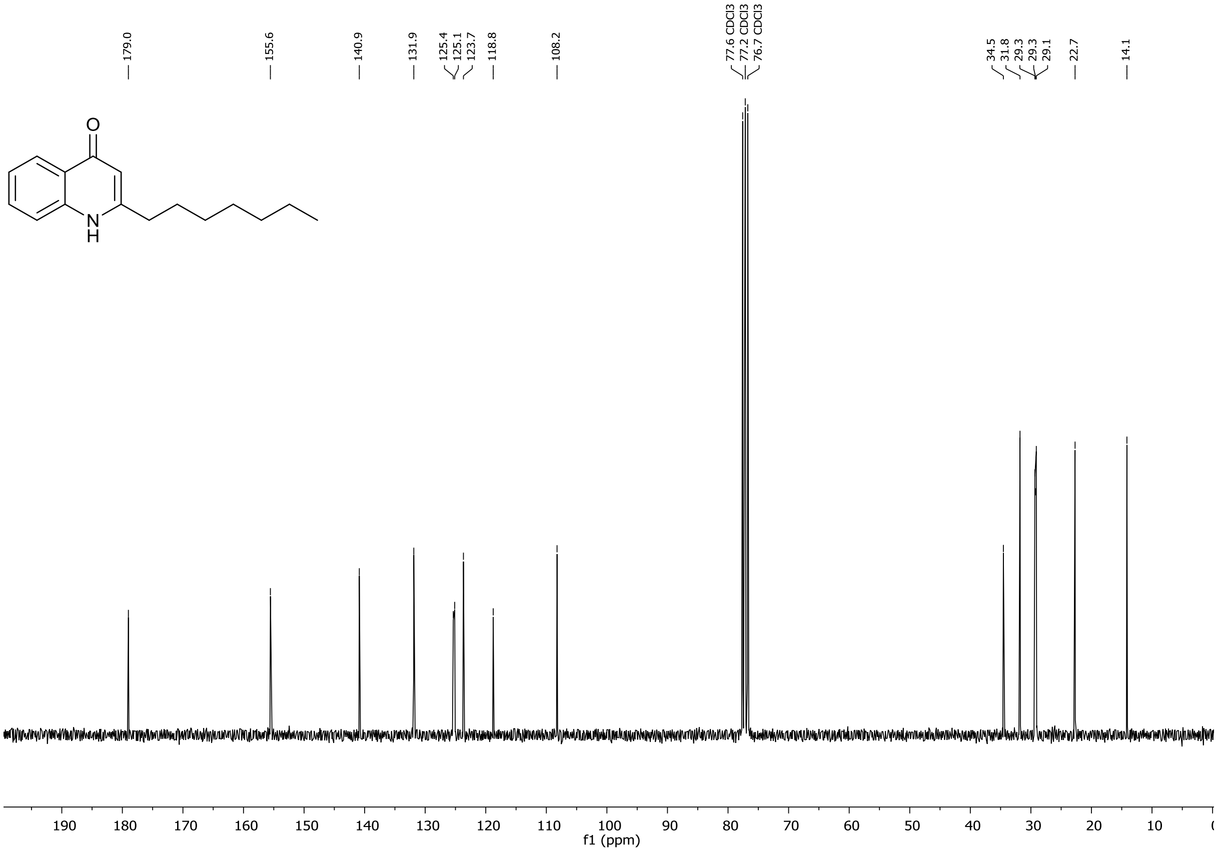

179.0
155.6
140.9
131.9
125.4
125.1
123.7
118.8
108.2
77.6 CDCl3
77.2 CDCl3
76.7 CDCl3
34.5
31.8
29.3
29.3
29.1
22.7
14.1
O
N
H
f1 (ppm)

NMe-HHQ (2)

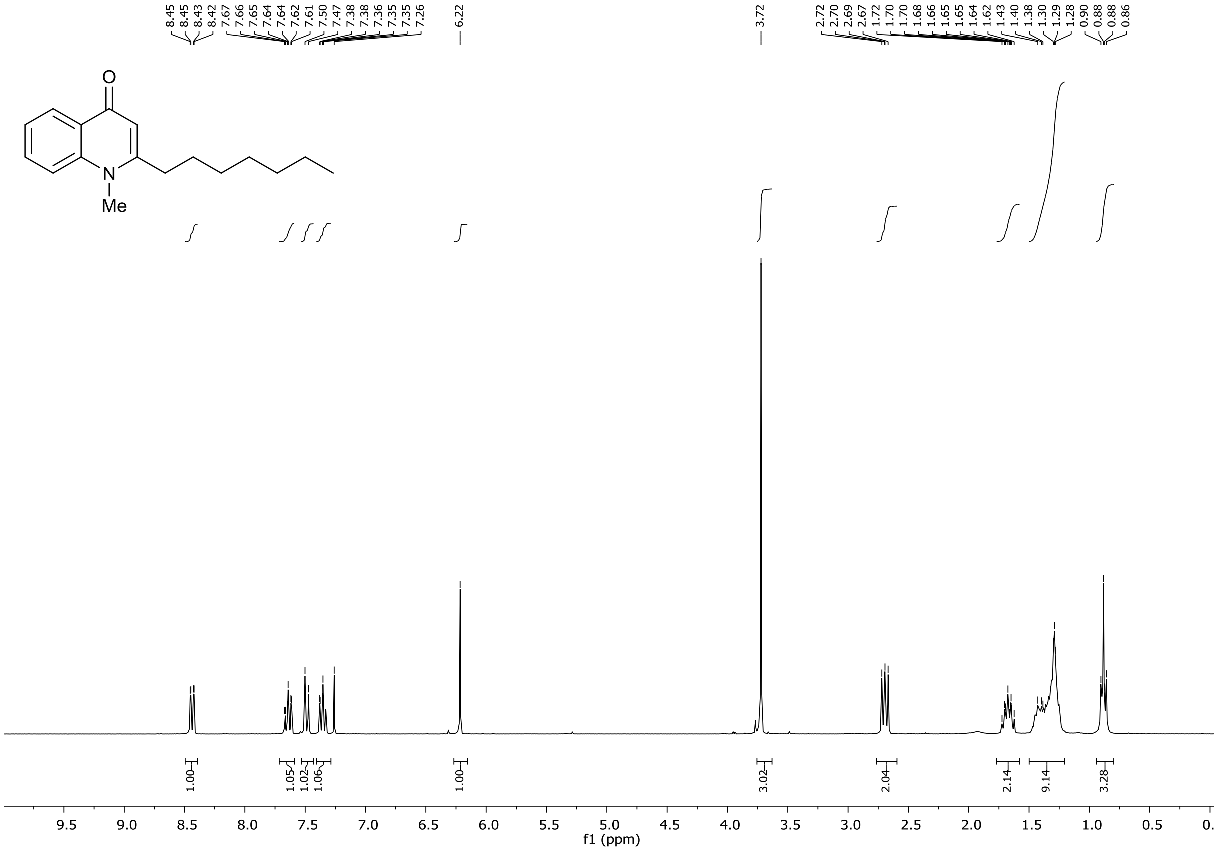
8.45 8.45 8.43 8.42 7.67 7.66 7.65 7.64 7.64 7.62 7.61 7.50 7.47 7.38 7.38 7.36 7.35 7.35 7.26
6.22
3.72
2.72 2.70 2.69 2.67 1.72 1.70 1.70 1.68 1.66 1.65 1.65 1.64 1.62 1.43 1.40 1.38 1.30 1.29 1.28 0.90 0.88 0.88 0.86
O
N
Me
1.00
1.05
1.02
1.06
1.00
3.02
2.04
2.14
9.14
3.28
9.5 9.0 8.5 8.0 7.5 7.0 6.5 6.0 5.5 5.0 4.5 4.0 3.5 3.0 2.5 2.0 1.5 1.0 0.5
f1 (ppm)

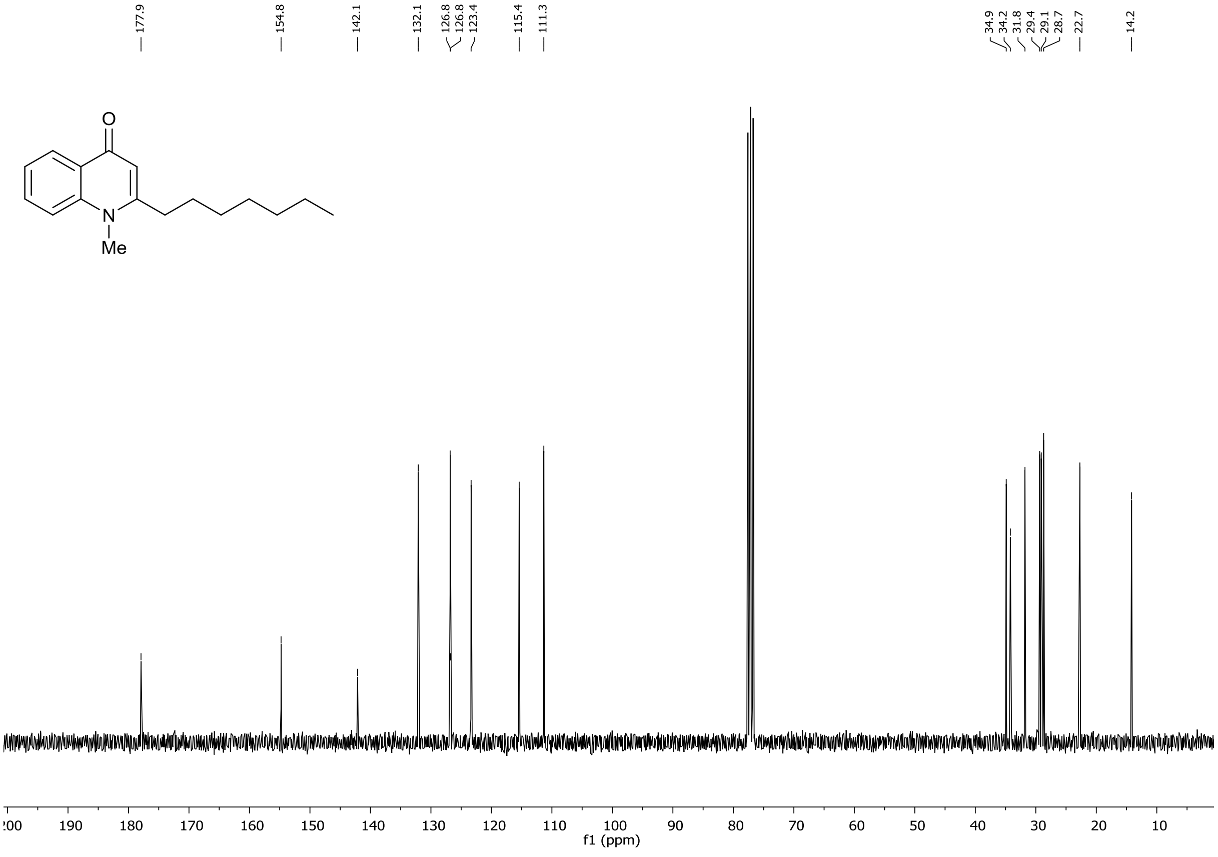
177.9
154.8
142.1
132.1 126.8 126.8 123.4
115.4 111.3
34.9 34.2 31.8 29.4 29.1 28.7
22.7
14.2
O
N
Me
190 180 170 160 150 140 130 120 110 100 90 80 70 60 50 40 30 20 10
f1 (ppm)

OMe-HHQ (3)

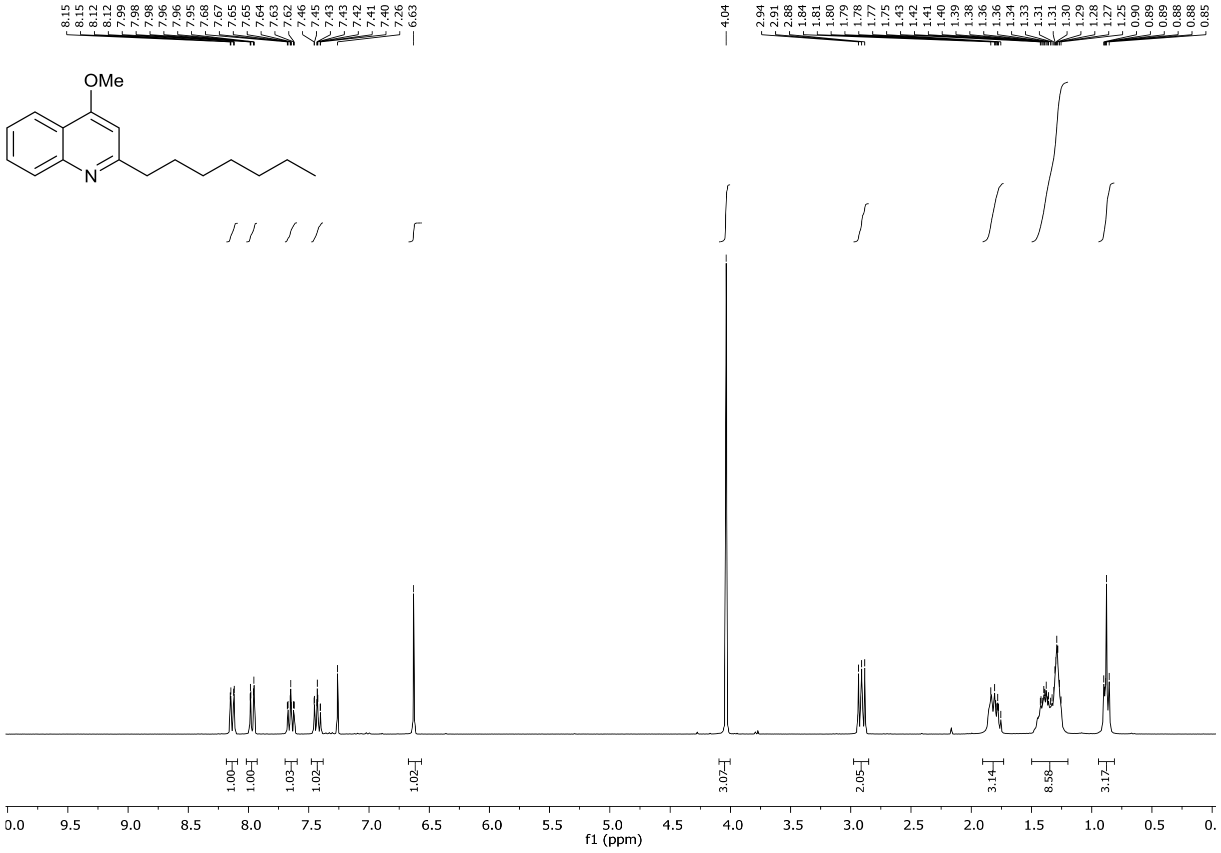

OMe
N
8.15 8.15 8.12 8.12 7.99 7.98 7.98 7.96 7.96 7.95 7.68 7.67 7.65 7.65 7.64 7.63 7.62 7.46 7.45 7.43 7.43 7.42 7.41 7.40 7.26 6.63
4.04
2.94 2.91 2.88 1.84 1.81 1.80 1.79 1.78 1.77 1.75 1.43 1.42 1.41 1.40 1.39 1.38 1.36 1.36 1.34 1.33 1.31 1.31 1.30 1.29 1.28 1.27 1.25 0.90 0.89 0.89 0.88 0.88 0.85
1.00 1.00 1.03 1.02 1.02 3.07 2.05 3.14 8.58 3.17
f1 (ppm)

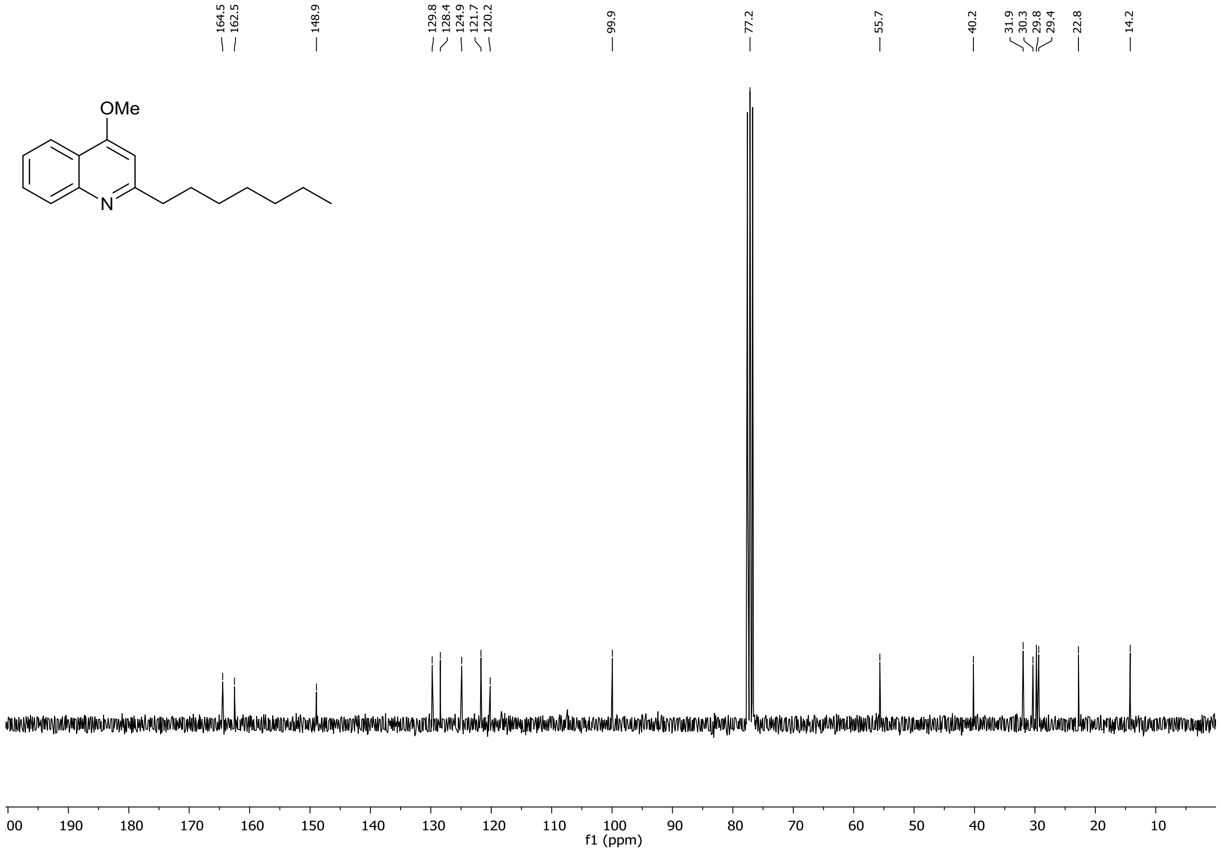

164.5 162.5 148.9 129.8 128.4 124.9 121.7 120.2 99.9 77.2 55.7 40.2 31.9 30.3 29.8 29.4 22.8 14.2
OMe
N
f1 (ppm)

Compound (4)

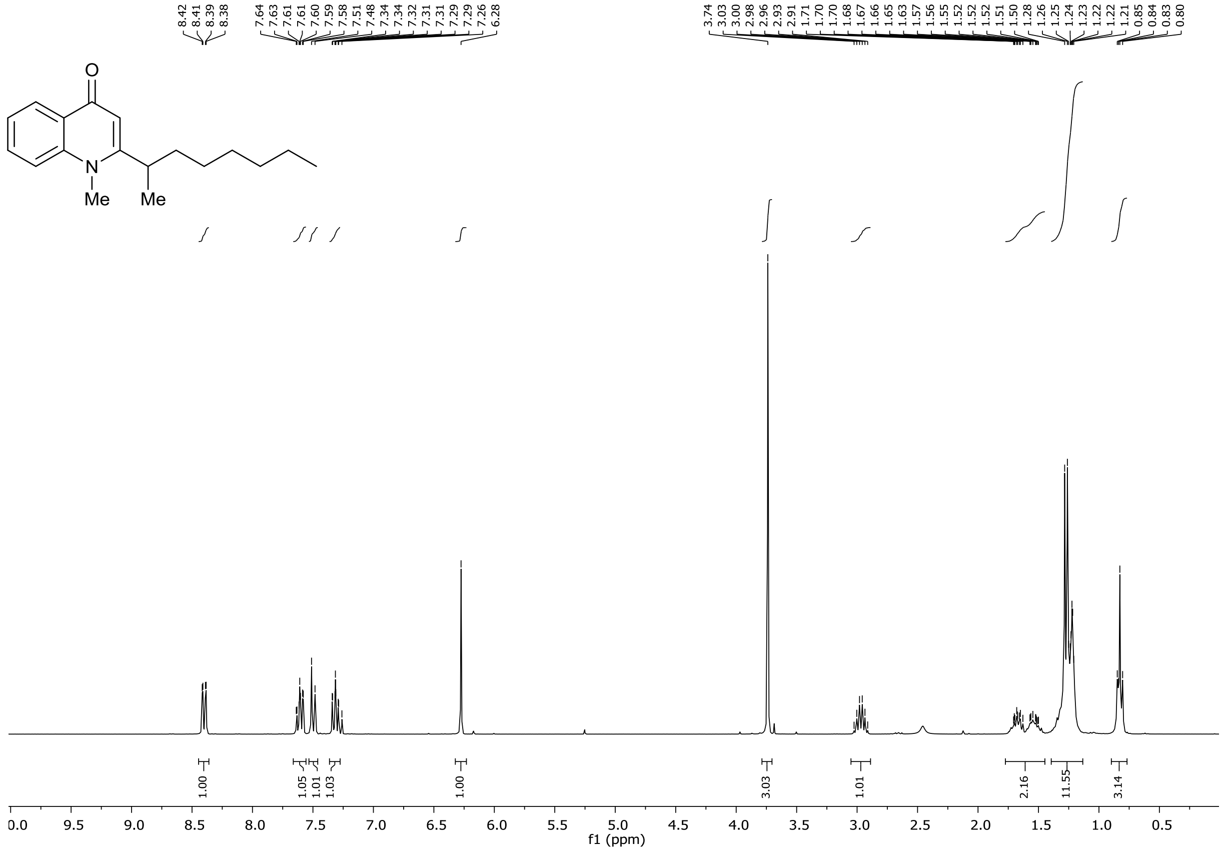

8.42 8.41 8.39 8.38
7.64 7.63 7.61 7.61 7.60 7.59 7.58 7.51 7.48 7.34 7.34 7.32 7.31 7.31 7.29 7.29 7.26 6.28
3.74 3.03 3.00 2.98 2.96 2.93 2.91 1.71 1.71 1.70 1.70 1.68 1.67 1.66 1.65 1.63 1.57 1.56 1.55 1.52 1.52 1.52 1.52 1.51 1.50 1.28 1.26 1.25 1.24 1.23 1.22 1.22 1.21 0.85 0.84 0.83 0.80
O
N
Me
Me
1.00 1.05 1.01 1.03 1.00 3.03 1.01 2.16 11.55 3.14
f1 (ppm)

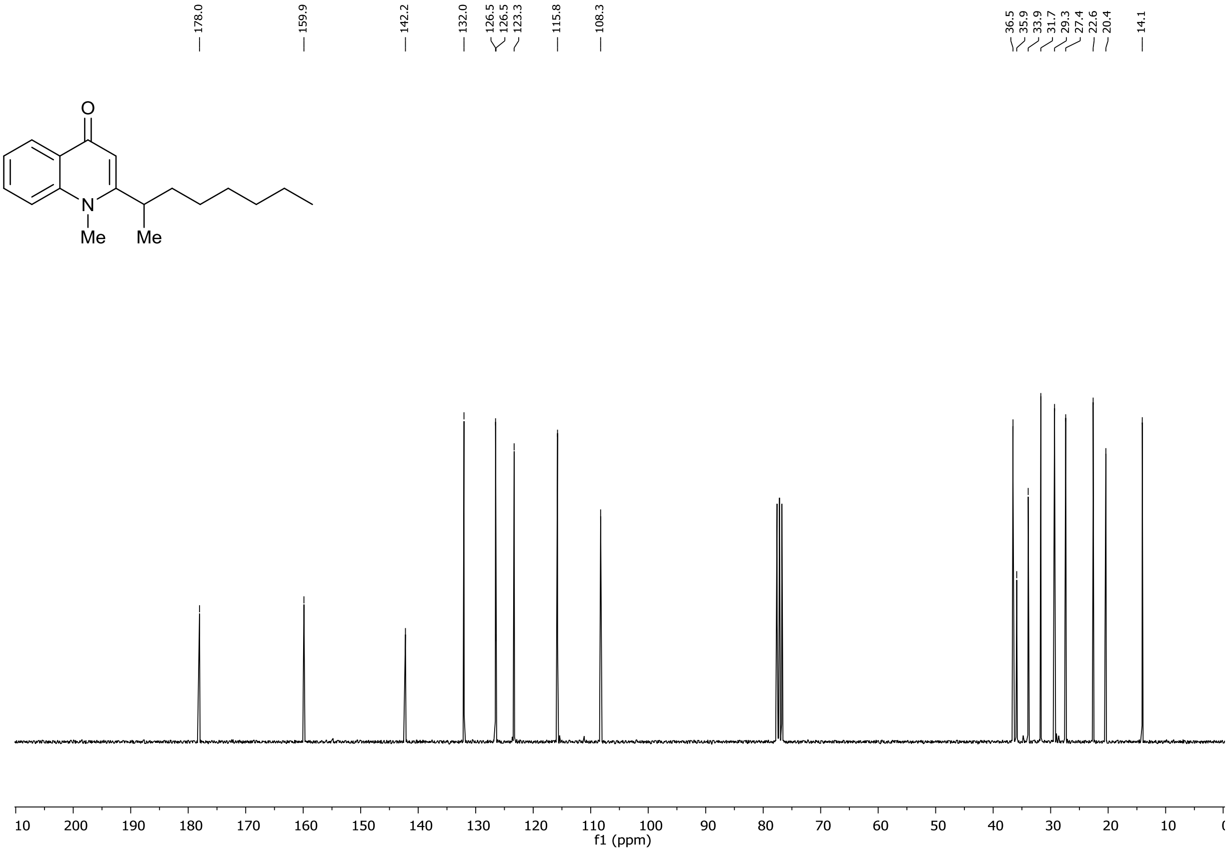

178.0 159.9 142.2 132.0 126.5 126.5 123.3 115.8 108.3
36.5 35.9 33.9 31.7 29.3 27.4 22.6 20.4 14.1
O
N
Me
Me
f1 (ppm)

HQNO (5)

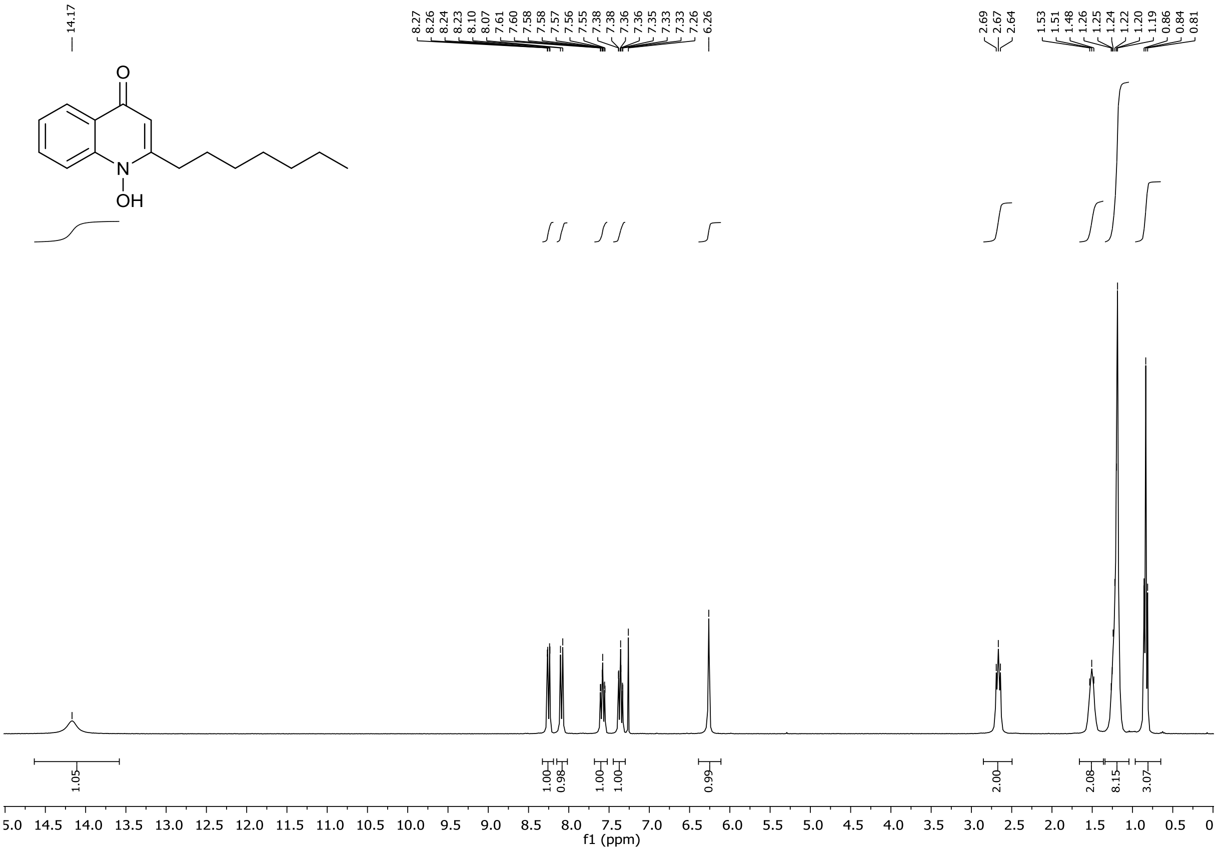

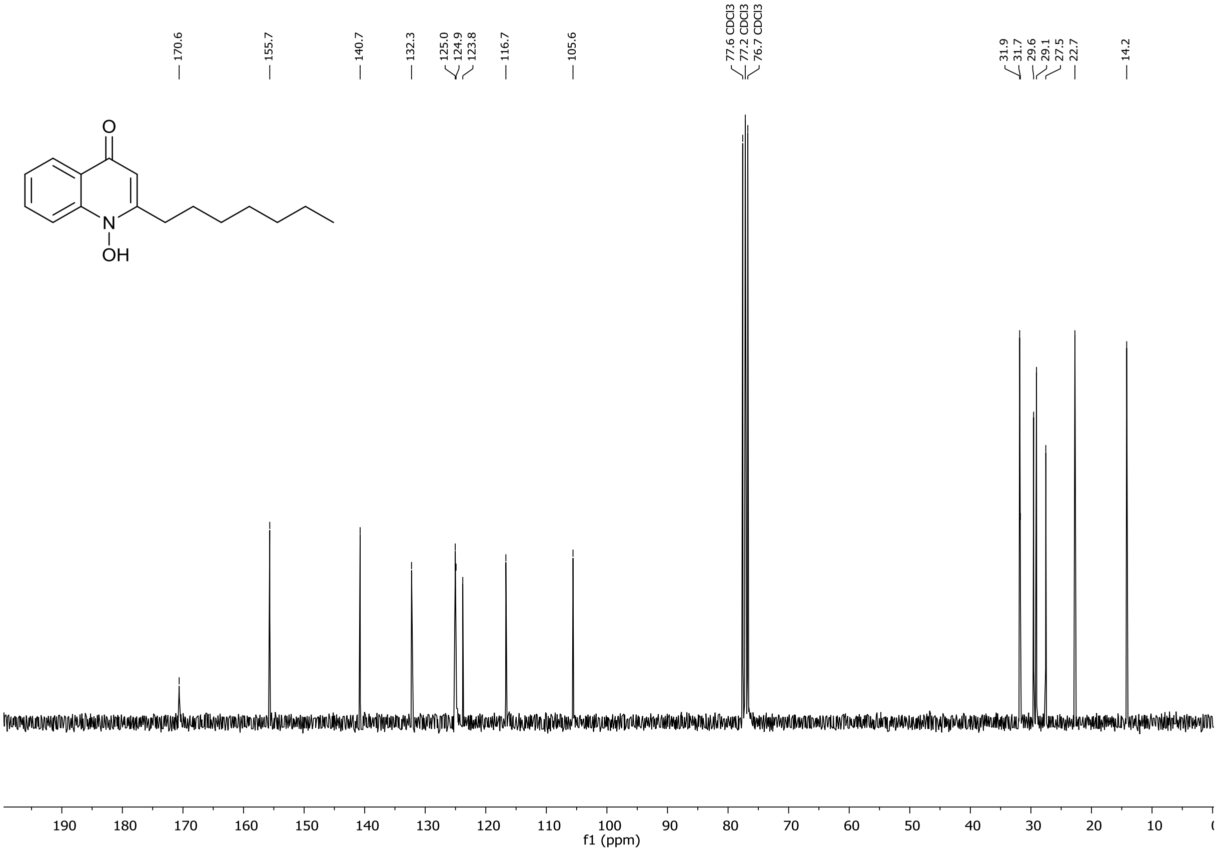

HMOQ (6)

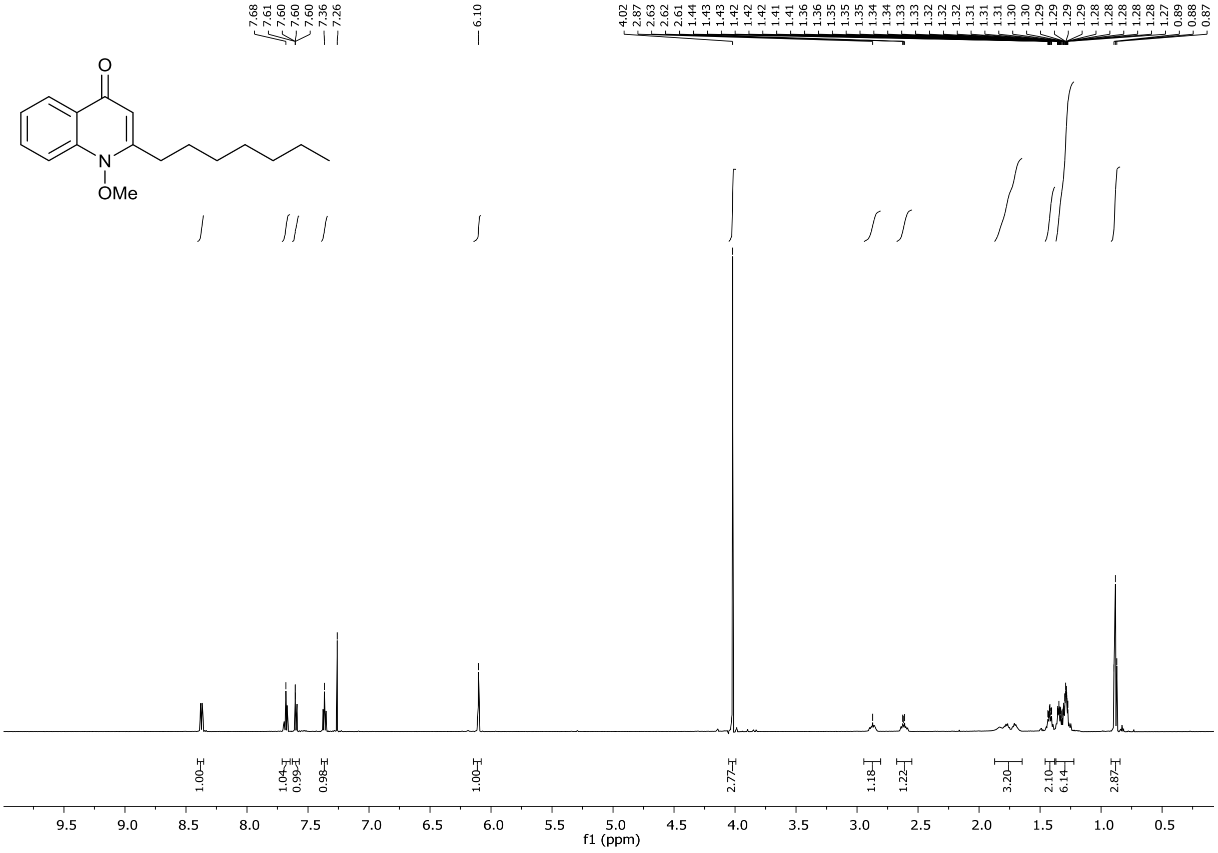
7.68
7.61
7.60
7.60
7.60
7.36
7.26
6.10
4.02
2.87
2.63
2.62
2.61
1.44
1.43
1.43
1.42
1.42
1.42
1.41
1.41
1.36
1.36
1.35
1.35
1.35
1.34
1.34
1.33
1.33
1.32
1.32
1.32
1.31
1.31
1.31
1.30
1.30
1.29
1.29
1.29
1.29
1.28
1.28
1.28
1.28
1.27
0.89
0.88
0.87
O
N
OMe
1.00
1.04
0.99
0.98
1.00
2.77
1.18
1.22
3.20
2.10
6.14
2.87
f1 (ppm)

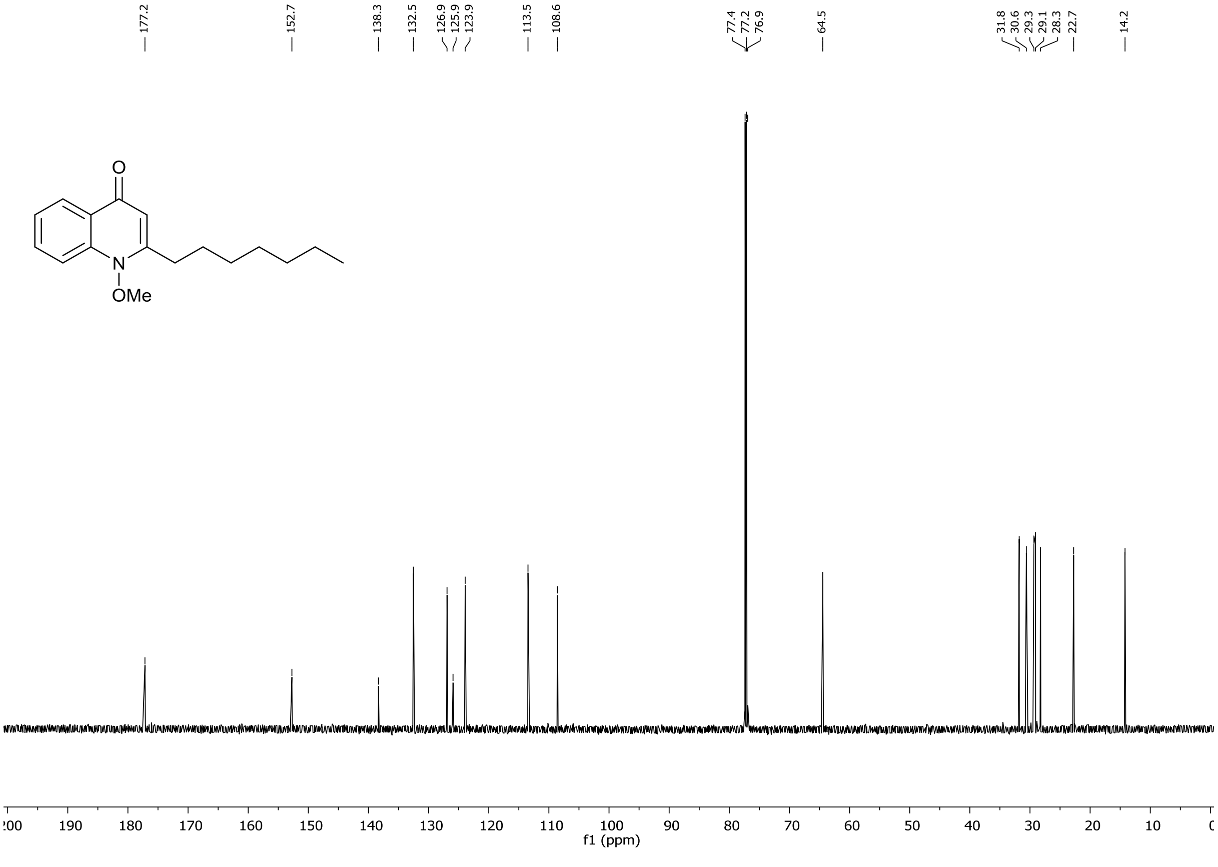
177.2
152.7
138.3
132.5
126.9
125.9
123.9
113.5
108.6
77.4
77.2
76.9
64.5
31.8
30.6
29.3
29.1
28.3
22.7
14.2
O
N
OMe
f1 (ppm)

4OMe-HQNO (7)

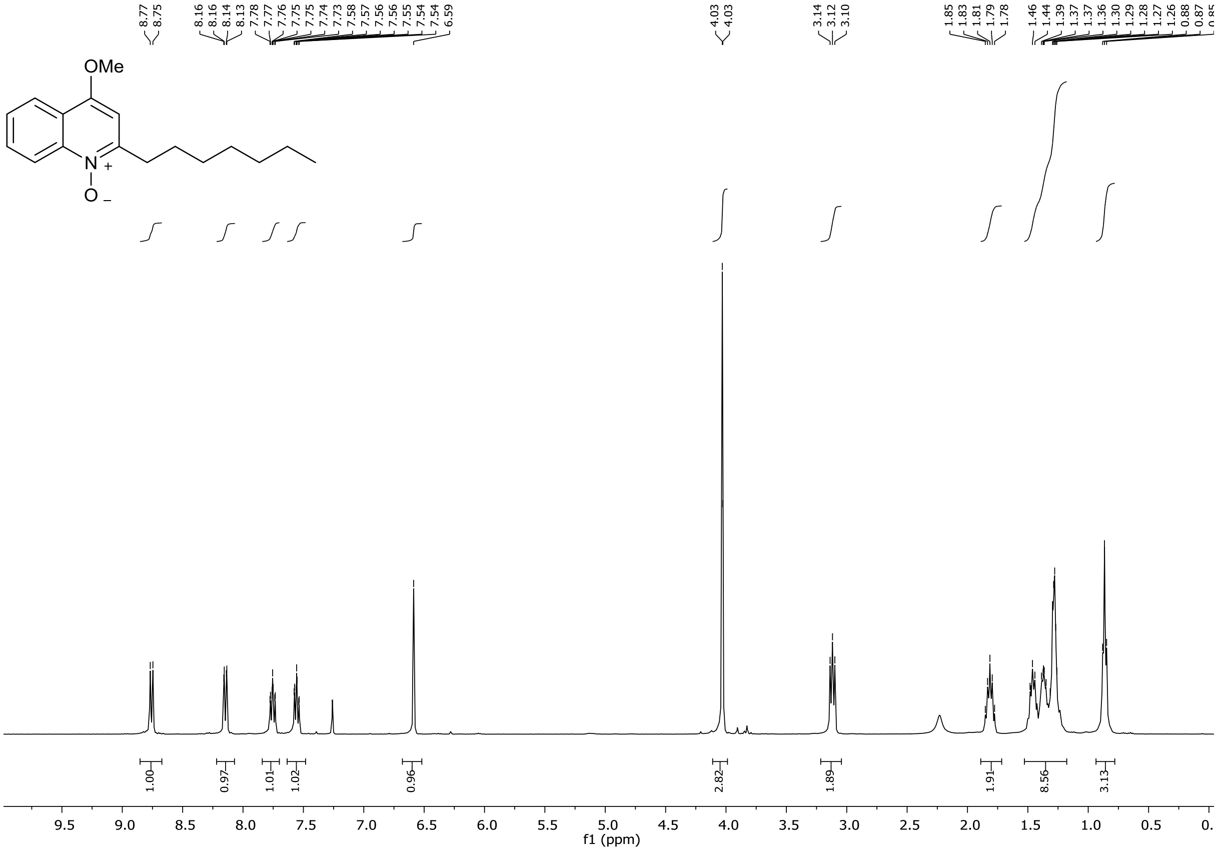

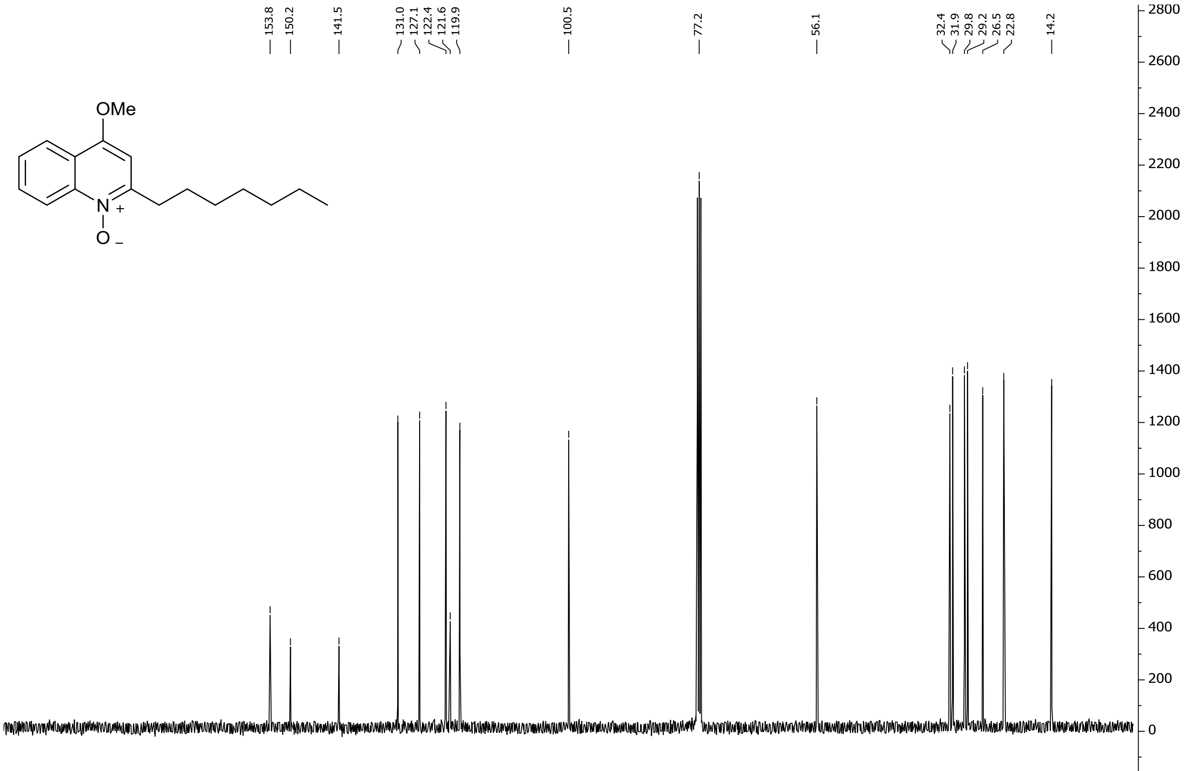

3I-HHQ (8)

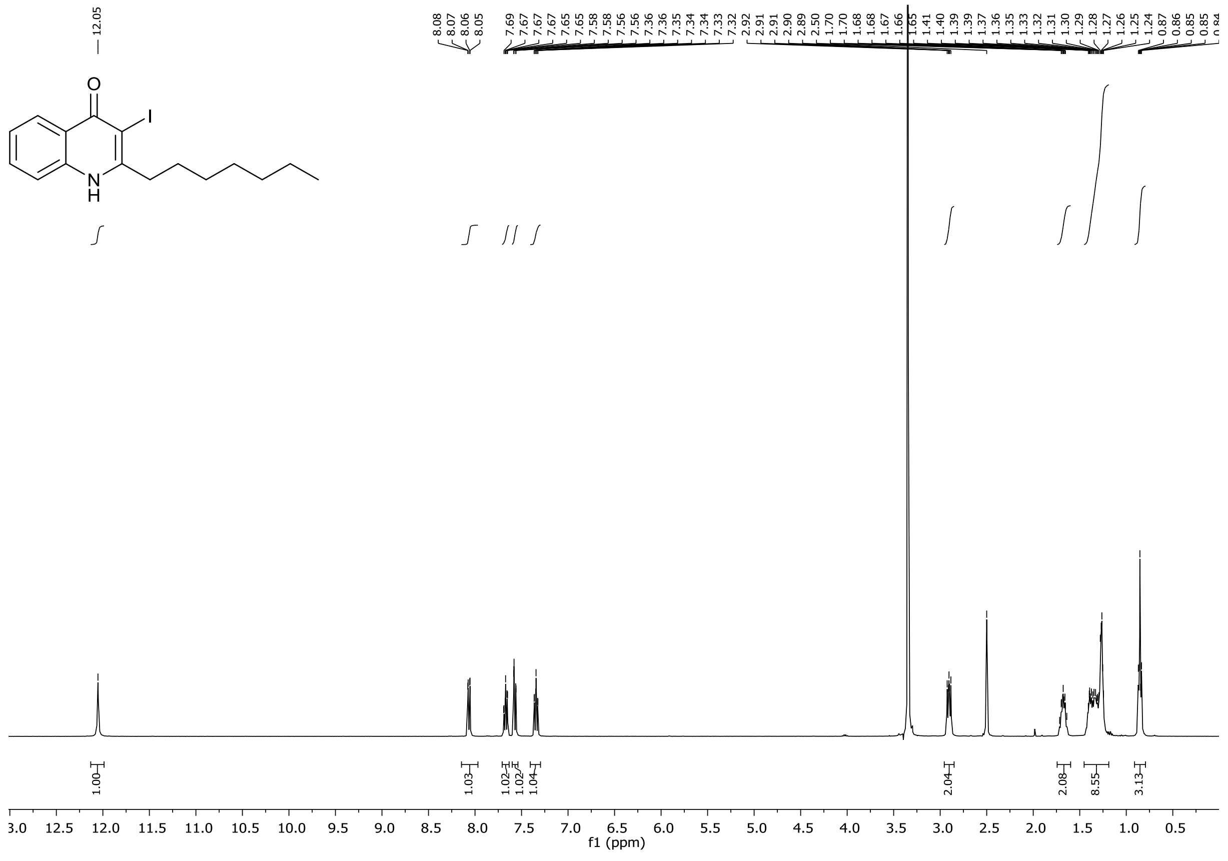

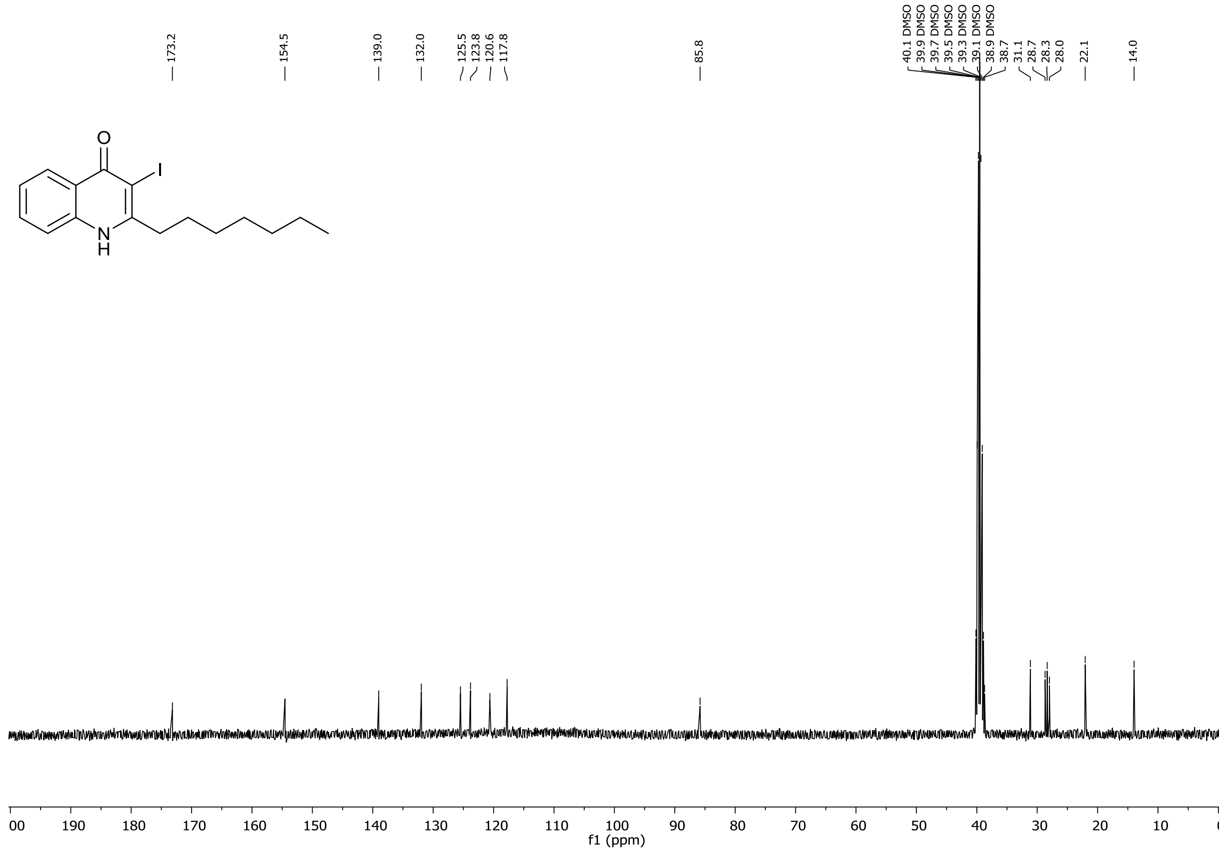

OMe-3I-HHQ (9)

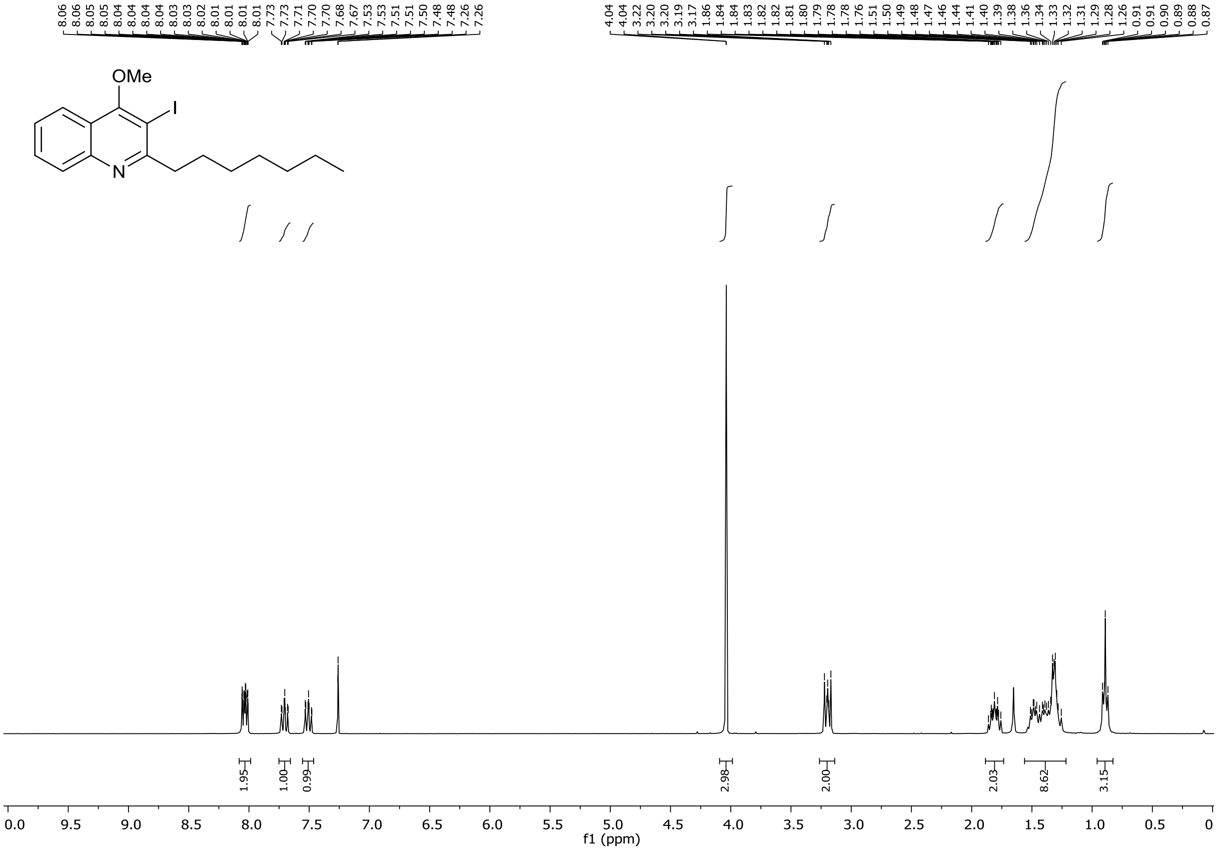
OMe
I
N
1.95
1.00
0.99
2.98
2.00
2.03
8.62
3.15
f1 (ppm)

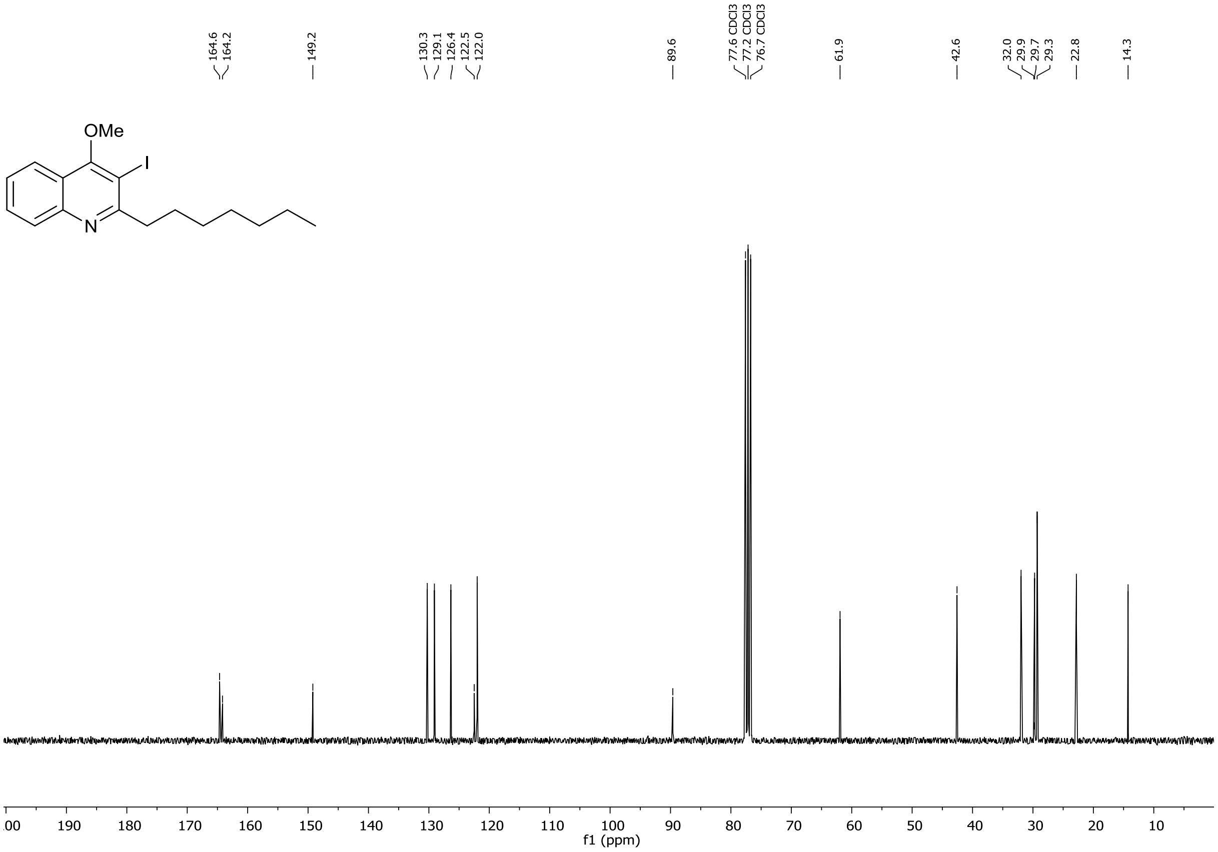
164.6
164.2
149.2
130.3
129.1
126.4
122.5
122.0
89.6
77.6 CDCl3
77.2 CDCl3
76.7 CDCl3
61.9
42.6
32.0
29.9
29.7
29.3
22.8
14.3
OMe
I
N
f1 (ppm)

NMe-3I-HHQ (10)

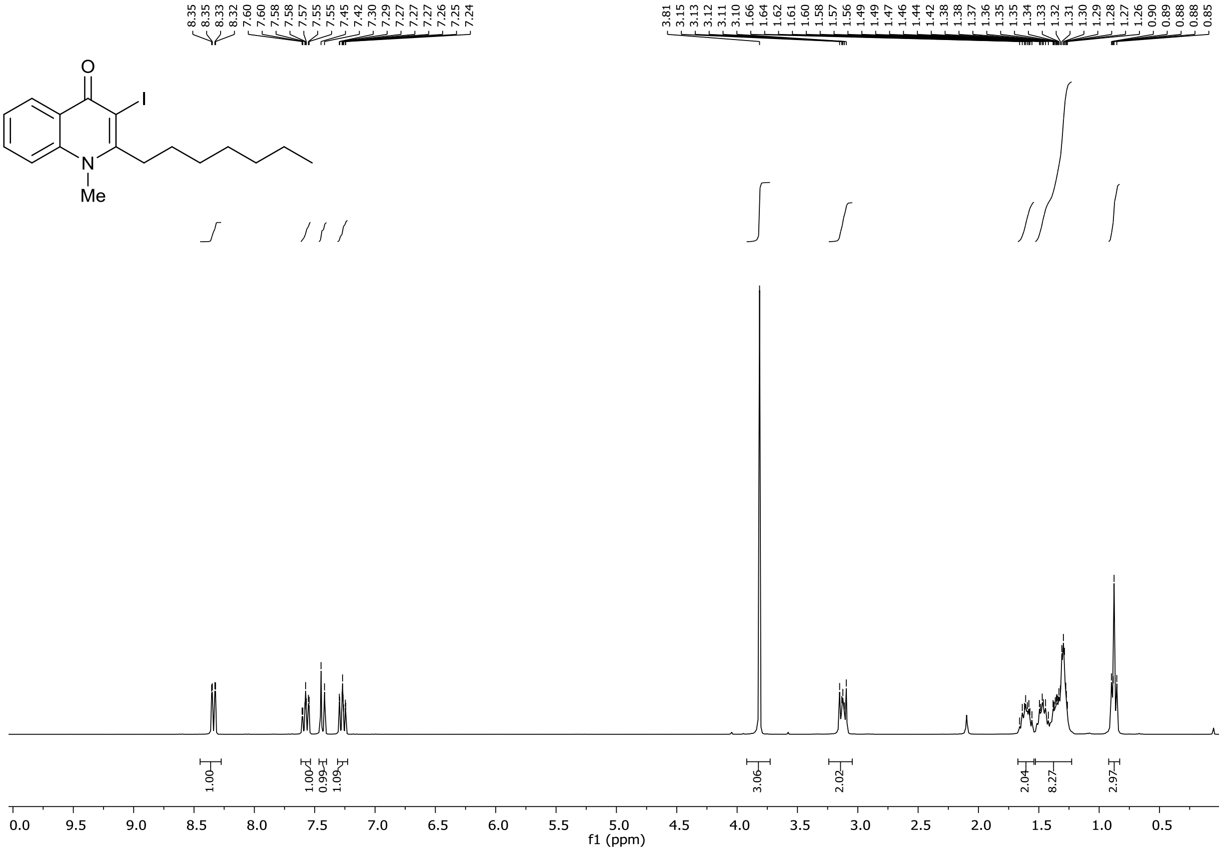
8.35 8.35 8.33 8.32 7.60 7.60 7.58 7.58 7.57 7.55 7.55 7.45 7.42 7.30 7.29 7.27 7.27 7.27 7.26 7.25 7.24
3.81 3.15 3.13 3.12 3.11 3.10 1.66 1.64 1.62 1.61 1.60 1.58 1.57 1.56 1.49 1.49 1.47 1.46 1.44 1.42 1.38 1.38 1.37 1.36 1.35 1.35 1.34 1.33 1.32 1.31 1.30 1.29 1.28 1.27 1.26 0.90 0.89 0.88 0.88 0.85
O
I
N
Me
1.00
1.00
0.99
1.09
3.06
2.02
2.04
8.27
2.97
f1 (ppm)

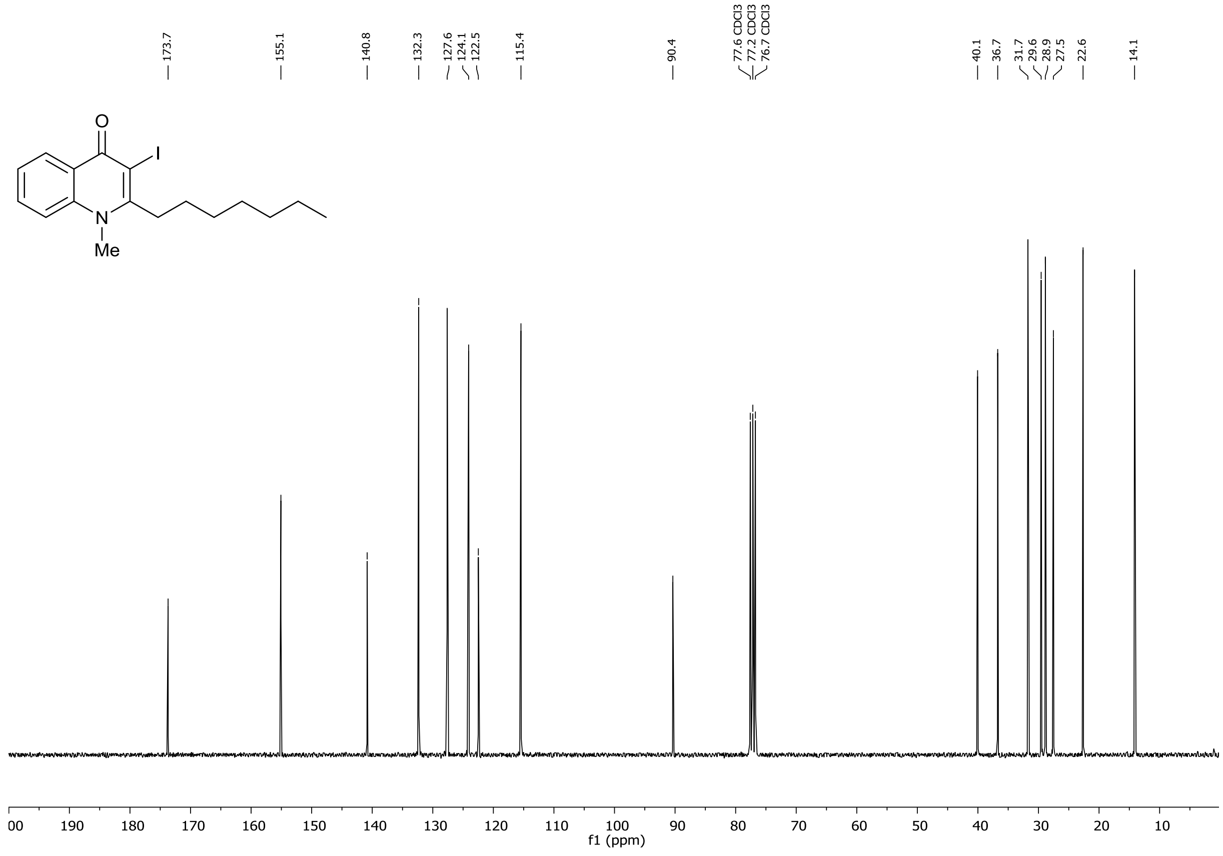
173.7
155.1
140.8
132.3
127.6
124.1
122.5
115.4
90.4
77.6 CDCl3
77.2 CDCl3
76.7 CDCl3
40.1
36.7
31.7
29.6
28.9
27.5
22.6
14.1
O
I
N
Me
f1 (ppm)

4OMe-PQS (11)

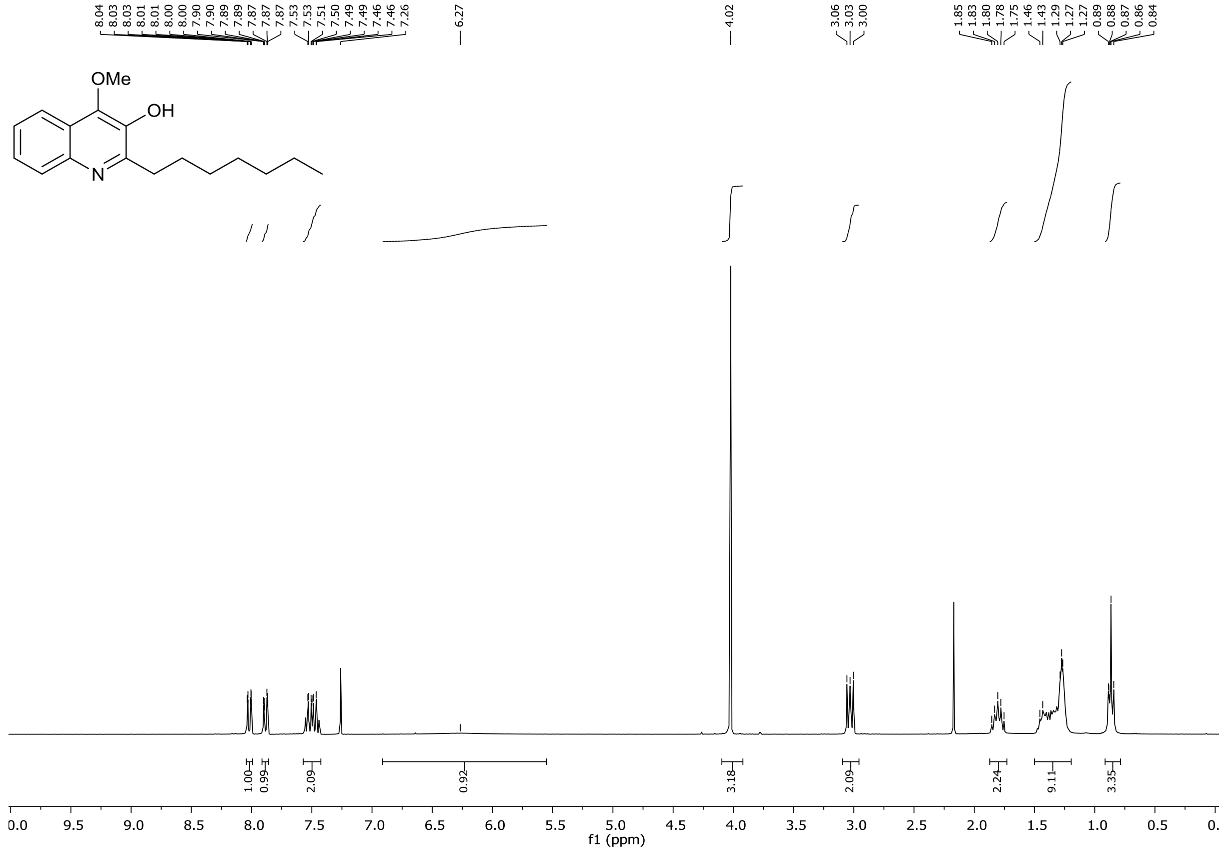

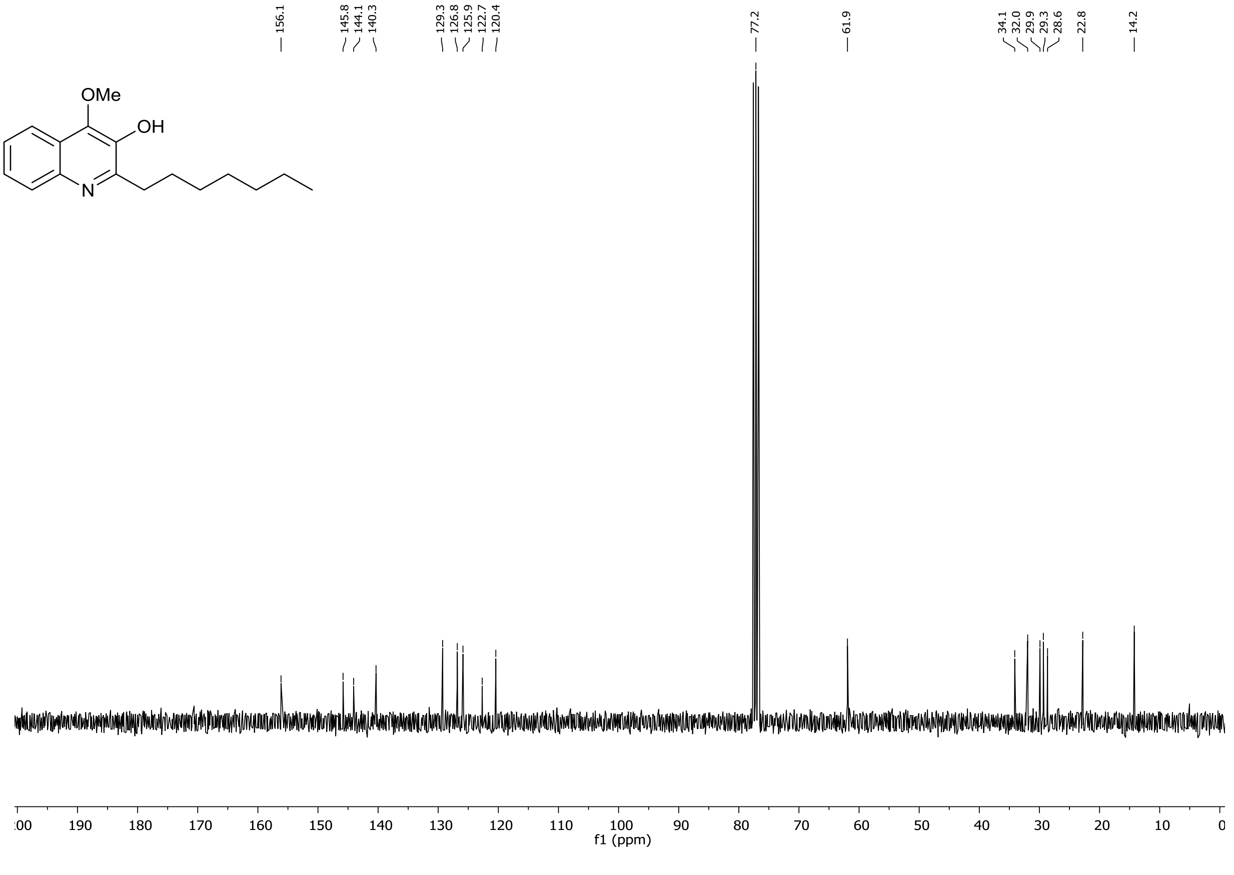

NMe-PQS (12)

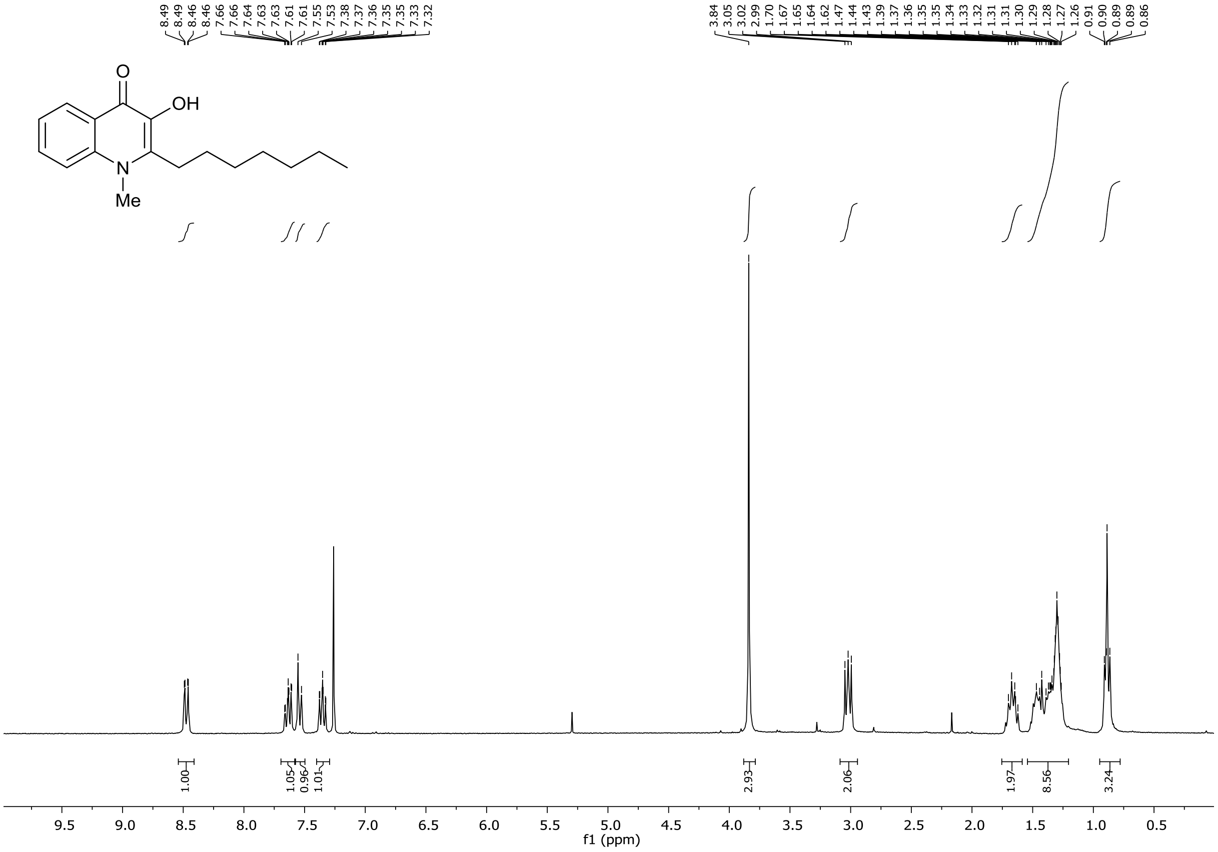
8.49 8.49 8.46 8.46 7.66 7.64 7.63 7.63 7.61 7.61 7.55 7.53 7.38 7.37 7.36 7.35 7.35 7.33 7.32
3.84 3.05 3.02 2.99 1.70 1.67 1.65 1.64 1.62 1.47 1.44 1.43 1.39 1.37 1.36 1.35 1.35 1.34 1.33 1.32 1.31 1.31 1.30 1.29 1.28 1.27 1.26 0.91 0.90 0.89 0.89 0.86
O
OH
N
Me
1.00
1.05
0.96
1.01
2.93
2.06
1.97
8.56
3.24
9.5 9.0 8.5 8.0 7.5 7.0 6.5 6.0 5.5 5.0 4.5 4.0 3.5 3.0 2.5 2.0 1.5 1.0 0.5
f1 (ppm)

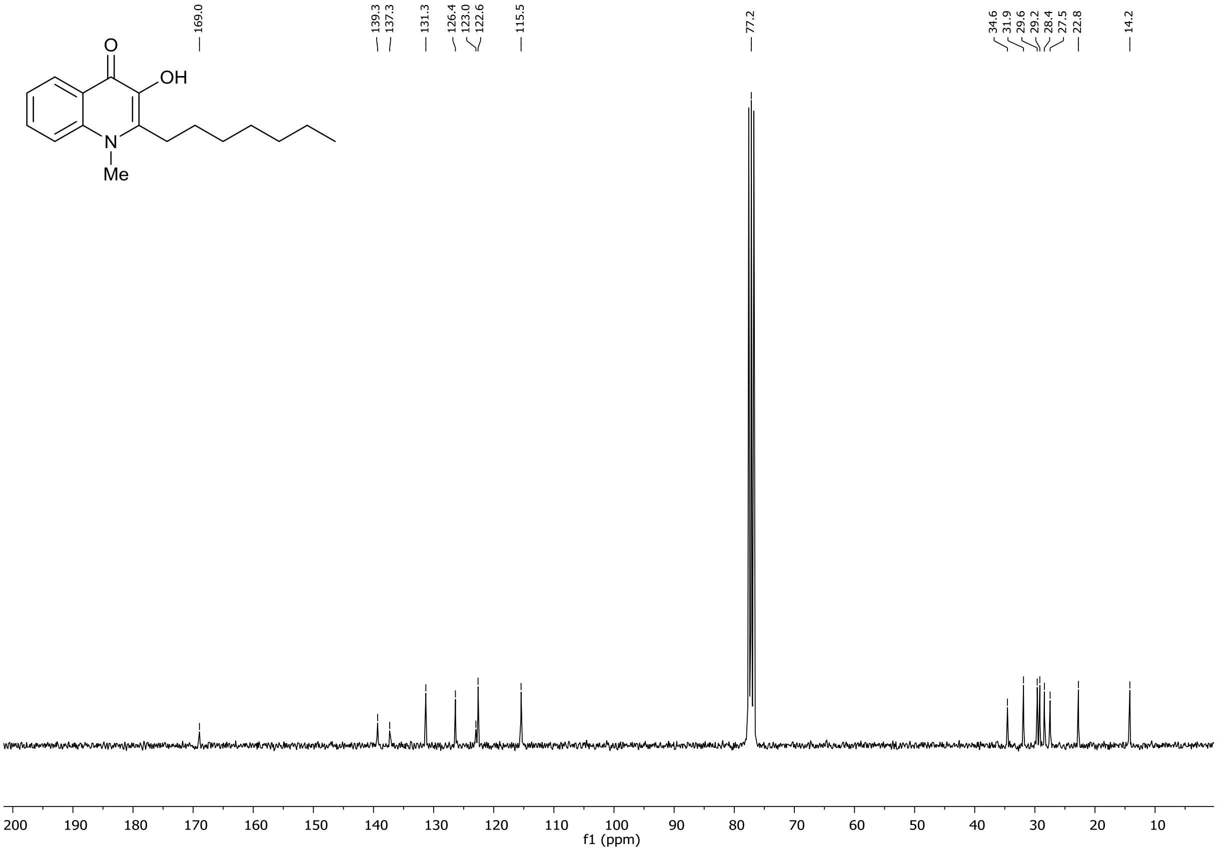
169.0
139.3 137.3 131.3 126.4 123.0 122.6 115.5
77.2
34.6 31.9 29.6 29.2 28.4 27.5 22.8 14.2
O
OH
N
Me
200 190 180 170 160 150 140 130 120 110 100 90 80 70 60 50 40 30 20 10
f1 (ppm)

5. Literature

[1] O'Toole, G. A. *J. Vis. Exp*. **2011,** *47,* e2437.

[2] Soh, E. Y.-C.; Chhabra, S. R.; Halliday, N.; Heeb, S.; Müller, C.; Birmes, F. S.; Fetzner, S.; Cámara, M.; Chan, K.-G.; Williams, P. *Environ. Microbiol*. **2015,** *17,* 4352-4365.

[3] Reynolds, G. A.; Hauser, C. R. *Org. Synth*. **1949,** *29,* 70.

[4] Reen, F. J.; Clarke, S. L.; Legendre, C.; McSweeney, C. M.; Eccles, K. S.; Lawrence, S. E.; O'Gara, F.; McGlacken, G. P. *Org. Biomol. Chem*. **2012,** *10,* 8903-8910.

[5] Woschek, A.; Mahout, M.; Mereiter, K.; Hammerschmidt, F. *Synthesis* **2007,** 1517-1522.

[6] Thierbach, S.; Birmes, F. S.; Letzel, M. C.; Hennecke, U.; Fetzner, S. *ACS Chem. Biol.* **2017,** *12,* 2305-2312.
